# Supplementary material for: Design, Synthesis, and Evaluation of Novel Indole Hybrid Chalcones and Their Antiproliferative and Antioxidant Activity
Source: Molecules. 2023 Sep 12;28(18):6583. doi: 10.3390/molecules28186583 (PMC10535268; doi:10.3390/molecules28186583)
Supplement: Supplementary file 1 [file molecules-28-06583-s001.zip › molecules-2614703-supplementary.pdf]

Article

Supplementary Materials: Design, Synthesis, and Evaluation of Novel Indole Hybrid Chalcones and Their Anticancer and Antioxidant activity

Zuzana Kudličková [1,*], Radka Michalková [2], Aneta Salayová [3], Marián Ksiažek [3], Mária Vilková [1], Slávka Bekešová [4] and Ján Mojžiš [2,*]

1 NMR Laboratory, Institute of Chemistry, Faculty of Science, Pavol Jozef Šafárik University, 040 01 Košice, Slovakia; maria.vilkova@upjs.sk
2 Department of Pharmacology, Faculty of Medicine, Pavol Jozef Šafárik University, 040 01 Košice, Slovakia; radka.michalkova@upjs.sk
3 Department of Chemistry, Biochemistry and Biophysics, University of Veterinary Medicine and Pharmacy in Košice, 041 81 Košice, Slovakia; aneta.salayova@uvlf.sk (A.S.); marian.ksiazek@student.uvlf.sk (M.K.)
4 Thermo Fisher Scientific, 821 09 Bratislava, Slovakia; slavka.bekesova@thermofisher.com
* Correspondence: zuzana.kudlickova@upjs.sk (Z.K.); jan.mojzis@upjs.sk (J.M.)

Table of contents:

Figures S1–S52. 1H NMR and 13C NMR Spectral Data of 11a, 11d-f, 12a-c, 13a-c, 14a-c, 17a-c, 18a-c, 19a-c 2–27
Figure S53. HR-MS fragmentation spectra for compound 11a...27

^{1}H NMR and ^{13}C NMR Spectral Data of 11a, 11d-f, 12a-c, 13a-c, 14a-c, 17a-c, 18a-c, 19a-c.

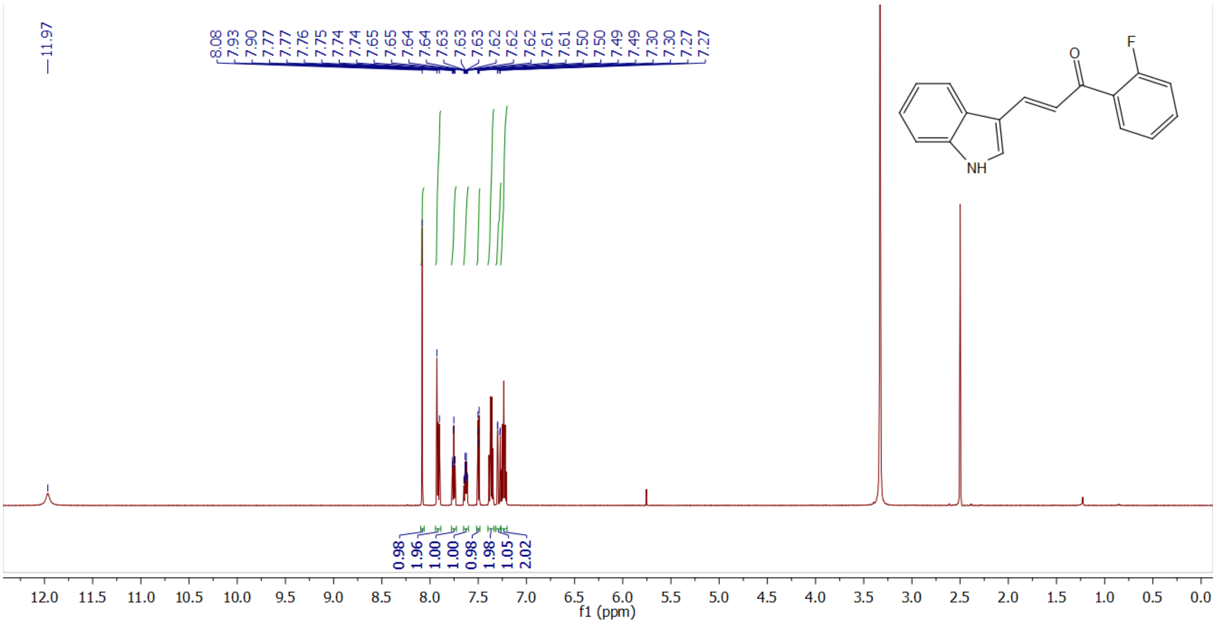

Figure S1: ^{1}H NMR spectra of compound **11a**

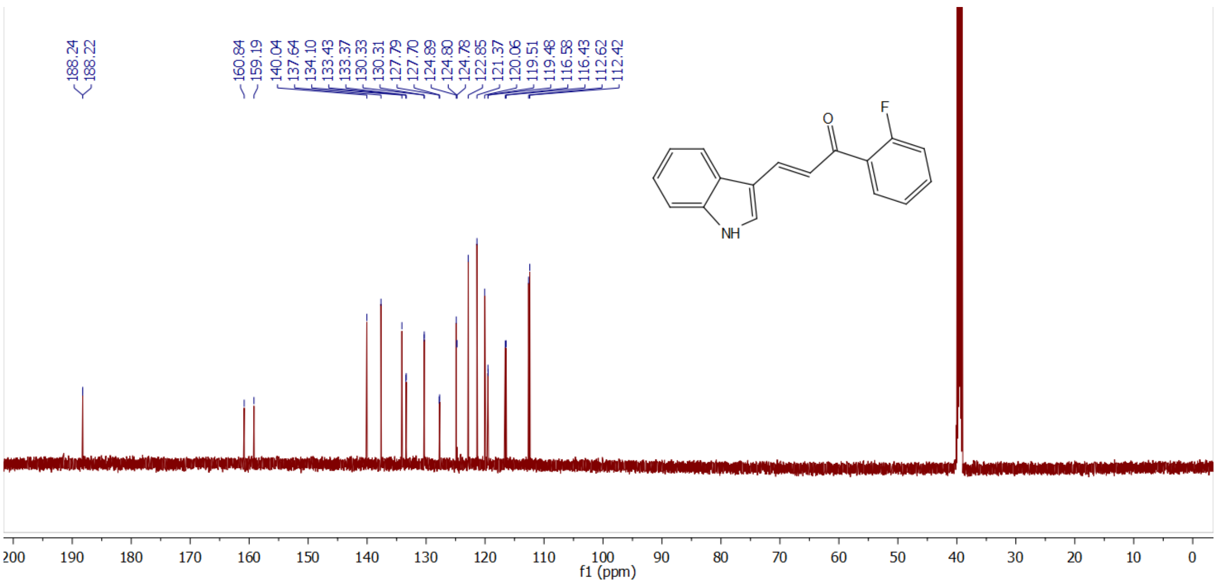

Figure S2: ^{13}C NMR spectra of compound **11a**

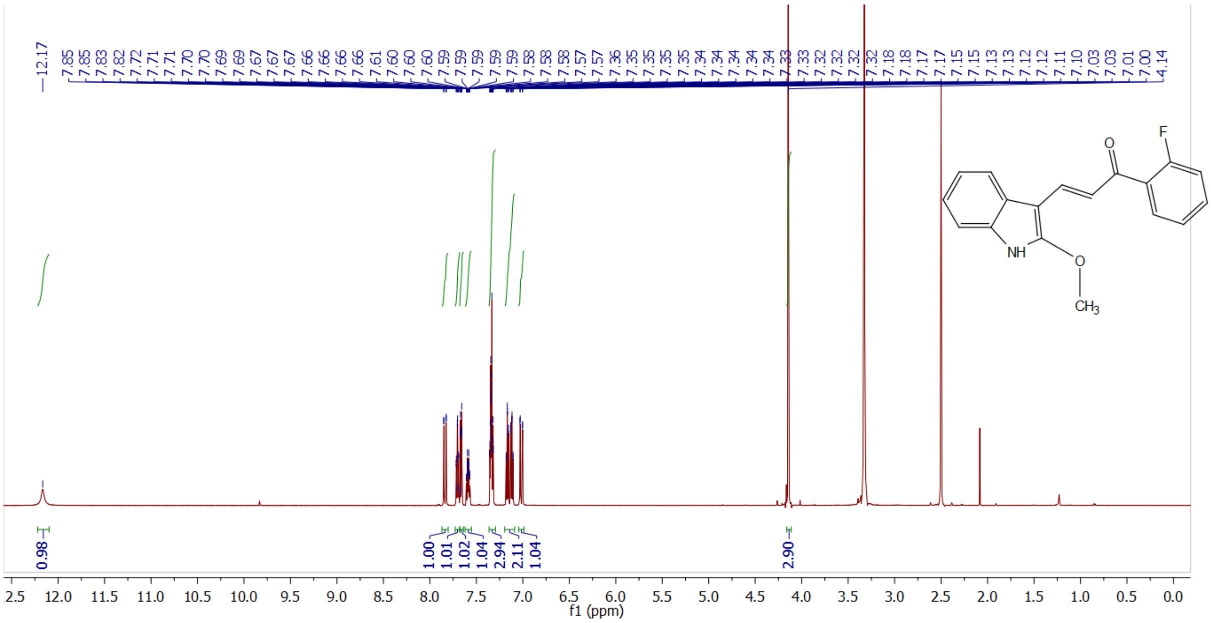

Figure S3: ^{1}H NMR spectra of compound **11d**

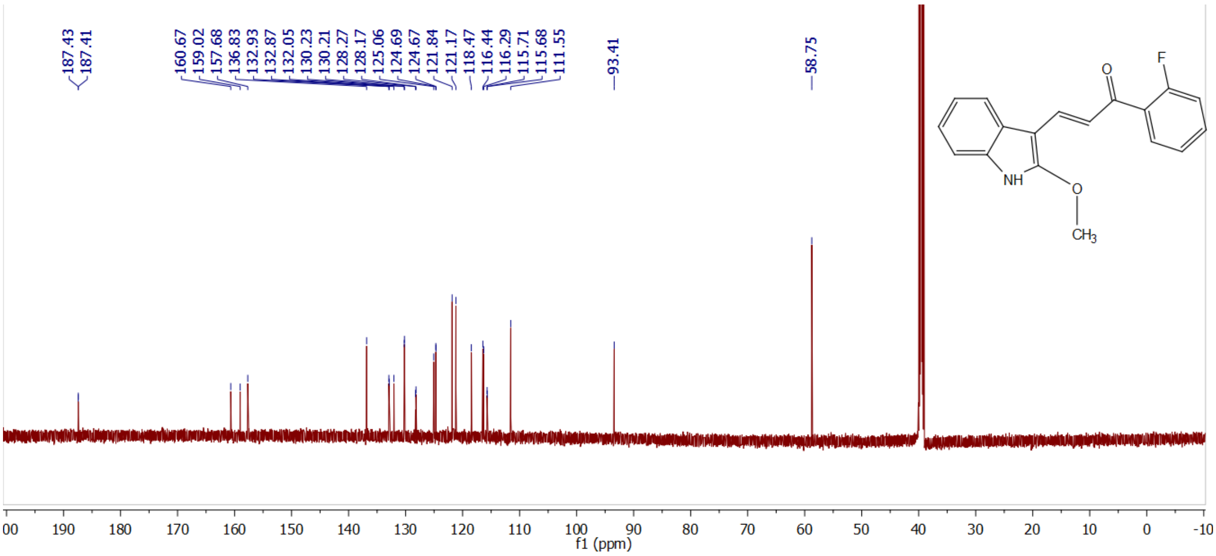

Figure S4: ^{13}C NMR spectra of compound **11d**

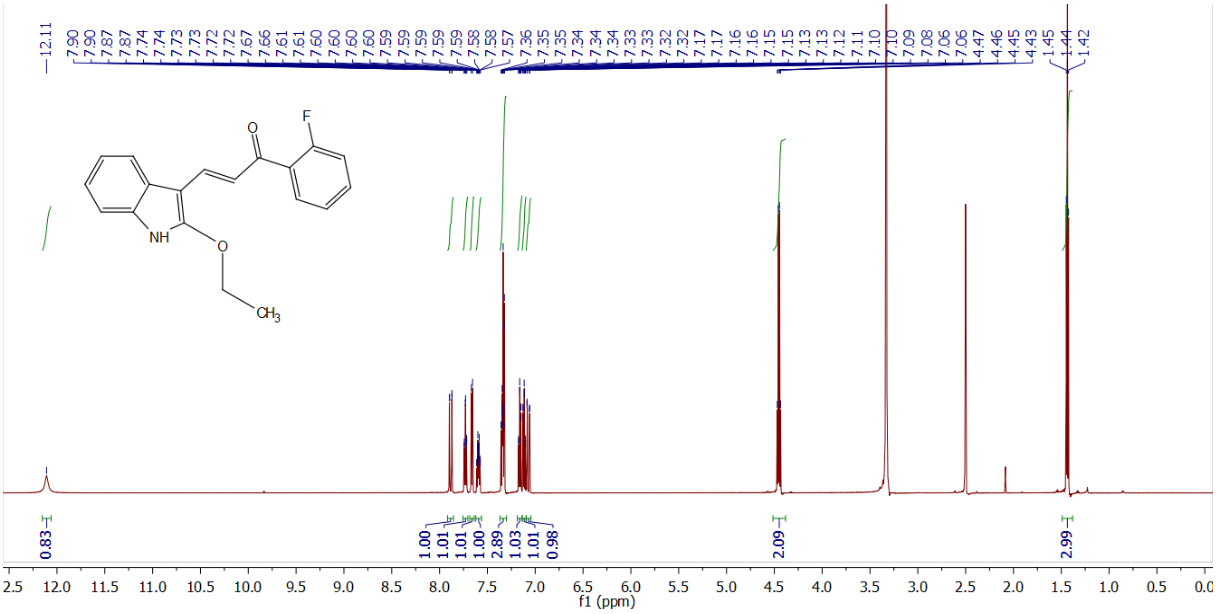

Figure S5: ^{1}H NMR spectra of compound **11e**

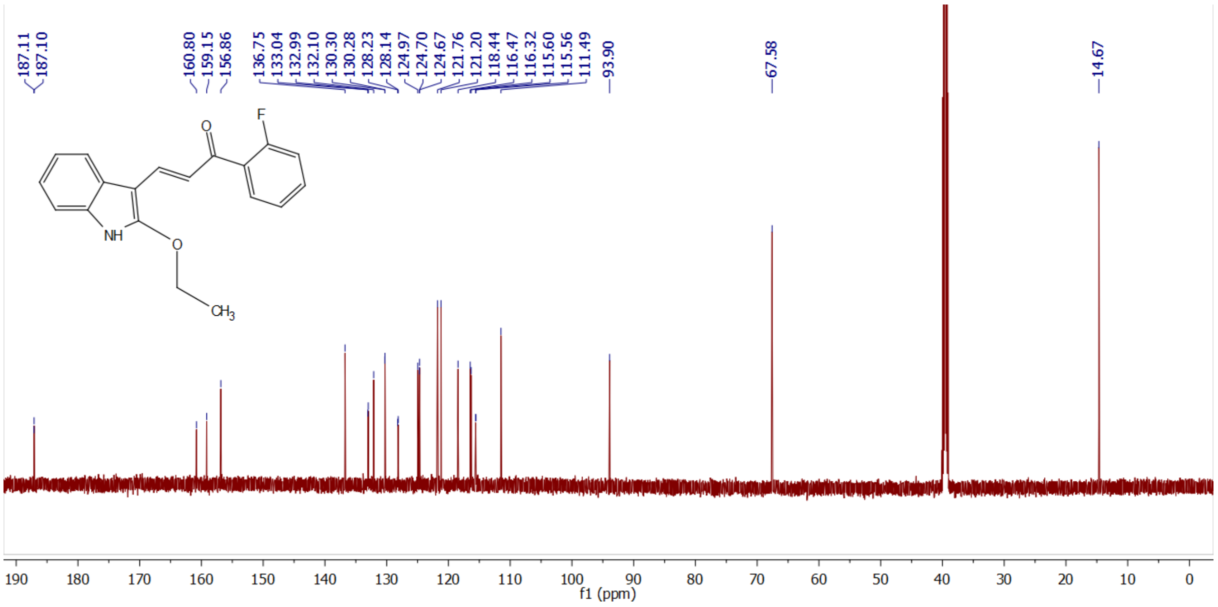

Figure S6: ^{13}C NMR spectra of compound **11e**

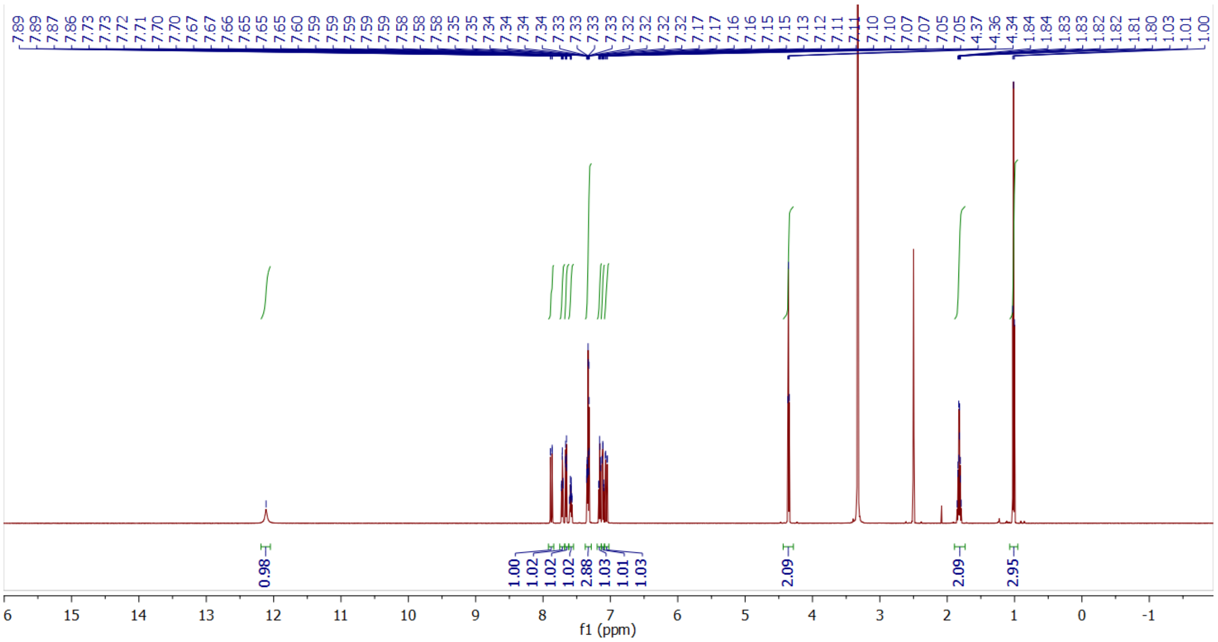

Figure S7: ^{1}H NMR spectra of compound **11f**

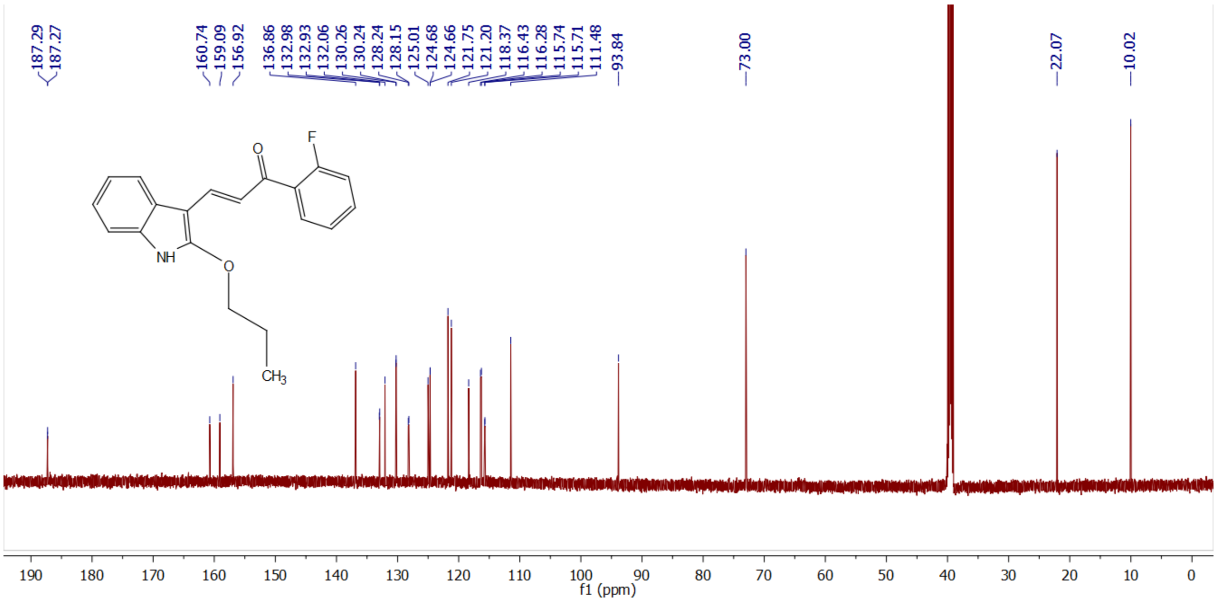

Figure S8: ^{13}C NMR spectra of compound **11f**

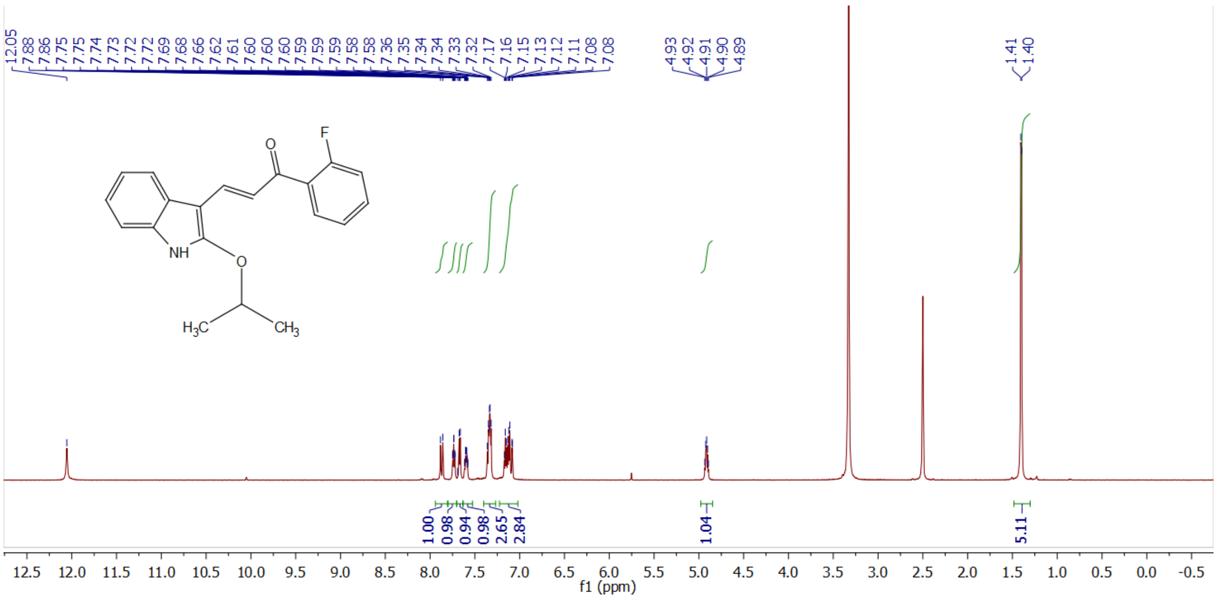

Figure S9: ^{1}H NMR spectra of compound **11g**

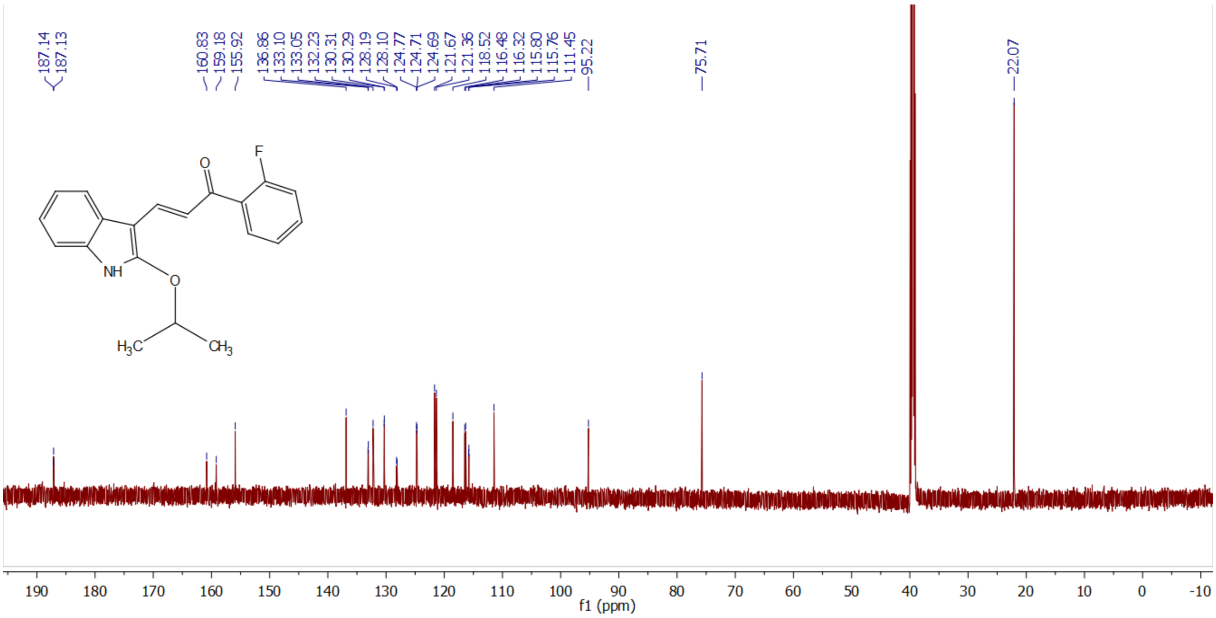

Figure S10: ^{13}C NMR spectra of compound **11g**

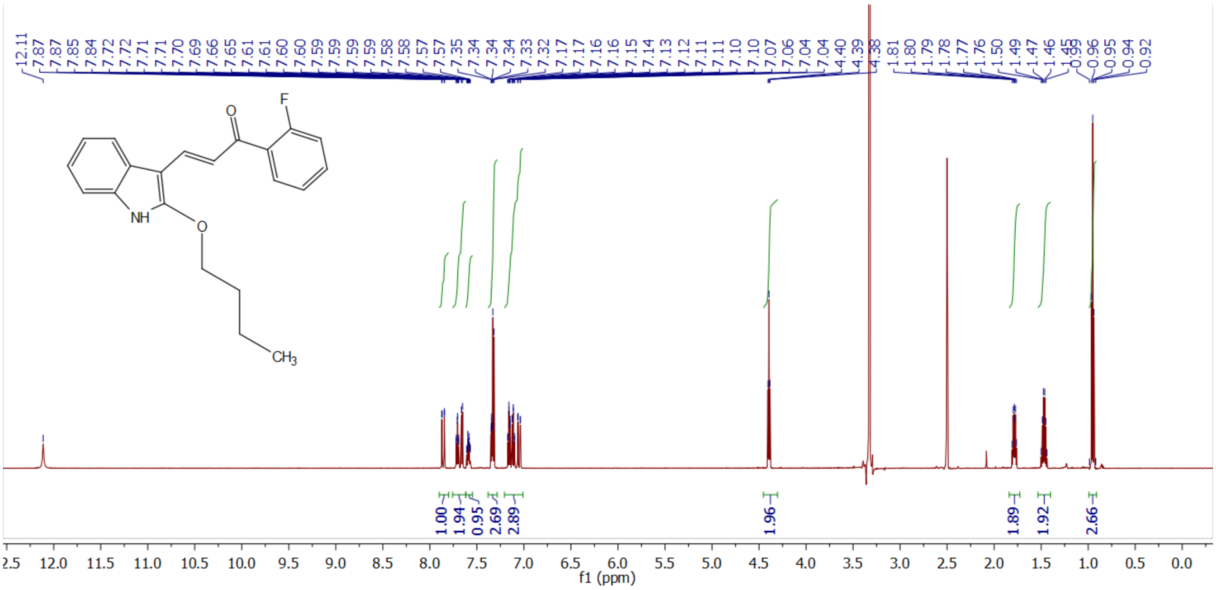

Figure S11: ^{1}H NMR spectra of compound **11h**

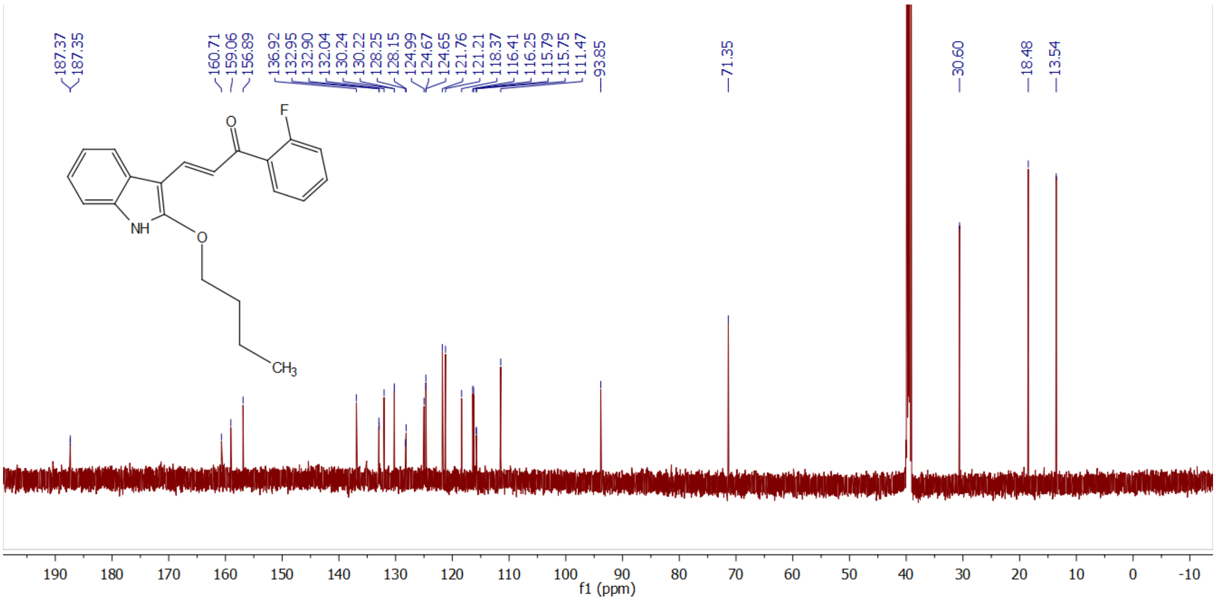

Figure S12: ^{13}C NMR spectra of compound **11h**

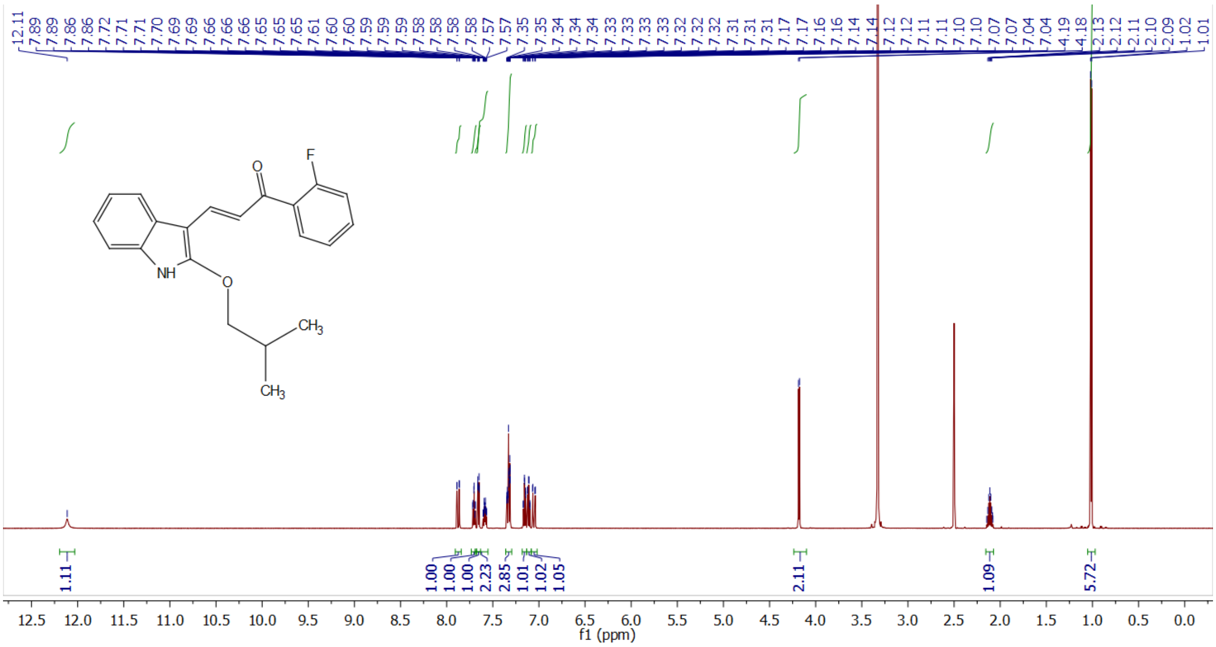

Figure S13: ^{1}H NMR spectra of compound **11i**

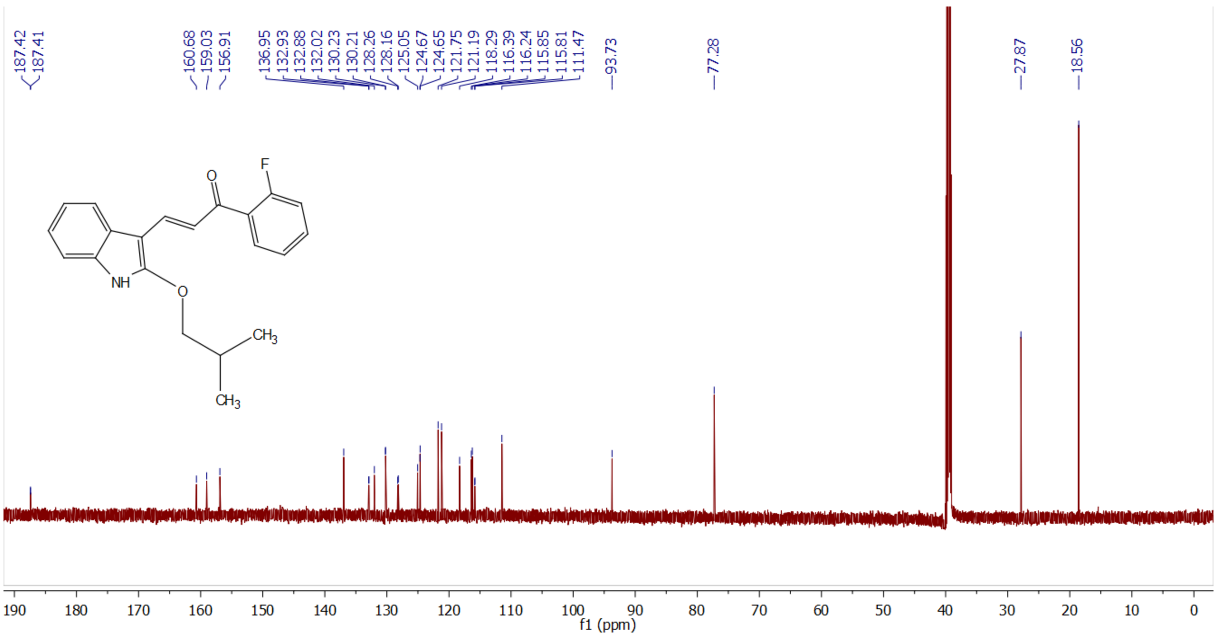

Figure S14: ^{13}C NMR spectra of compound **11i**

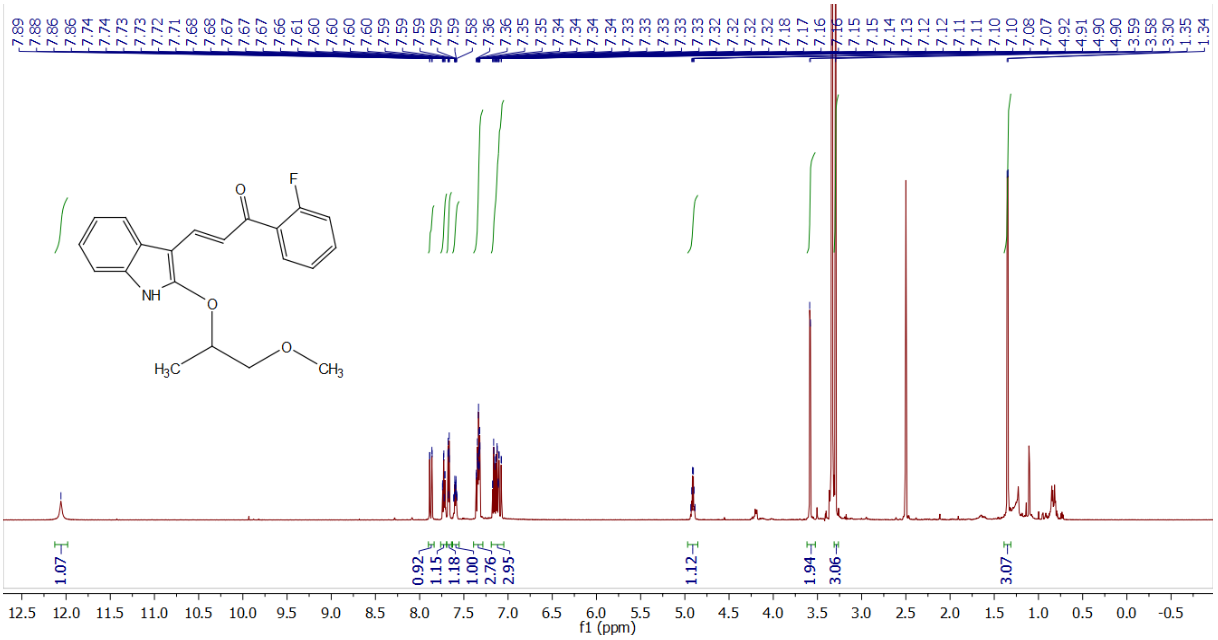

Figure S15: ^{1}H NMR spectra of compound **11j**

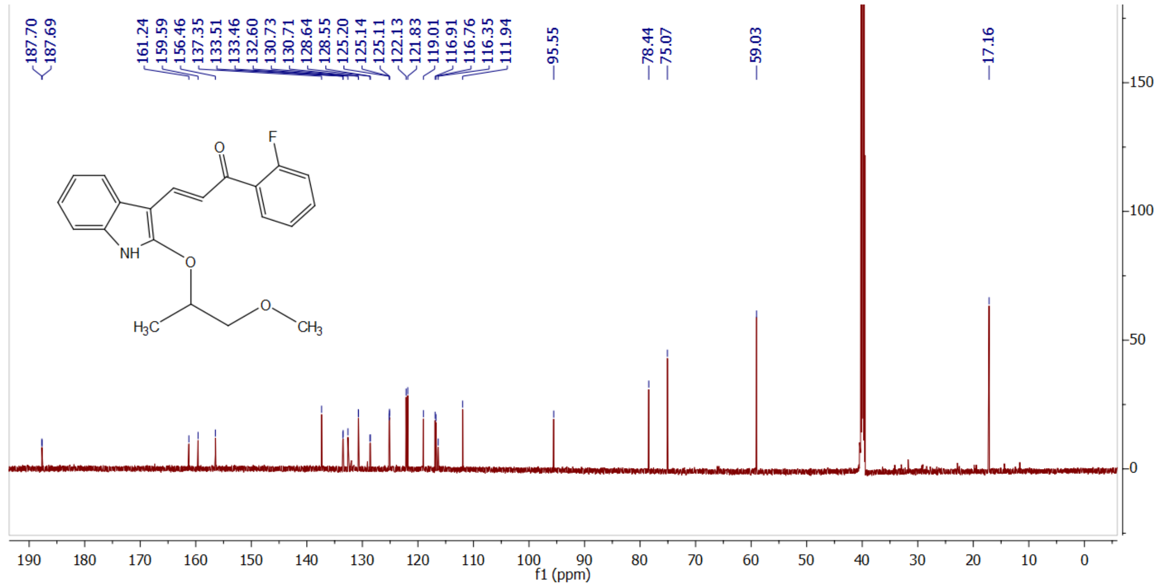

Figure S16: ^{13}C NMR spectra of compound **11j**

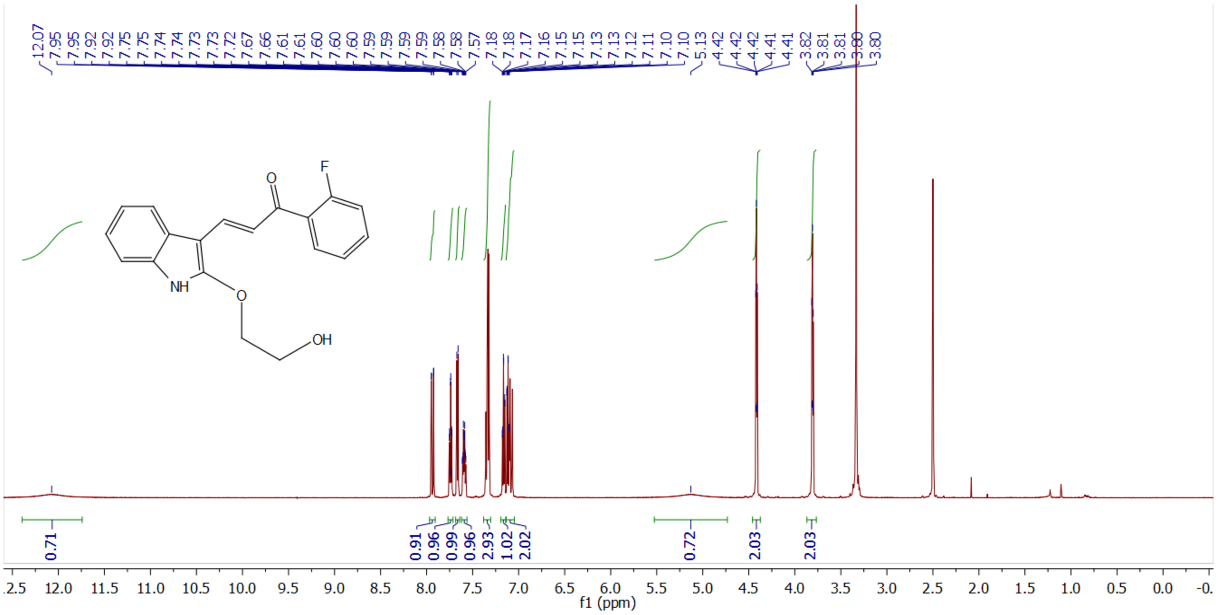

Figure S17: ^{1}H NMR spectra of compound **11k**

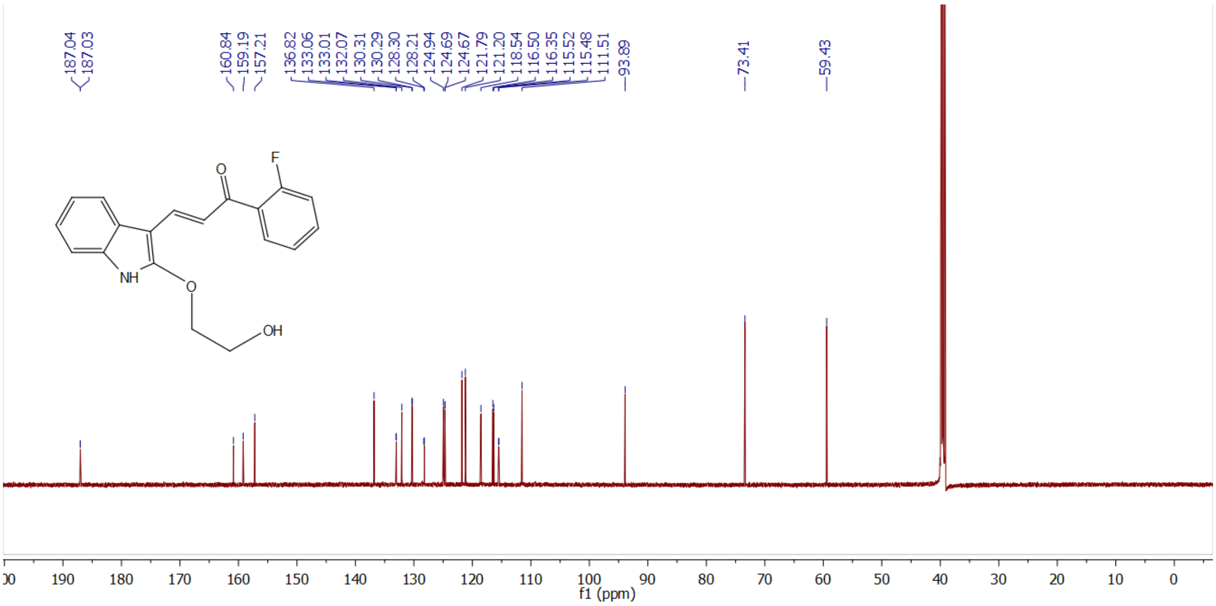

Figure S18: ^{13}C NMR spectra of compound **11k**

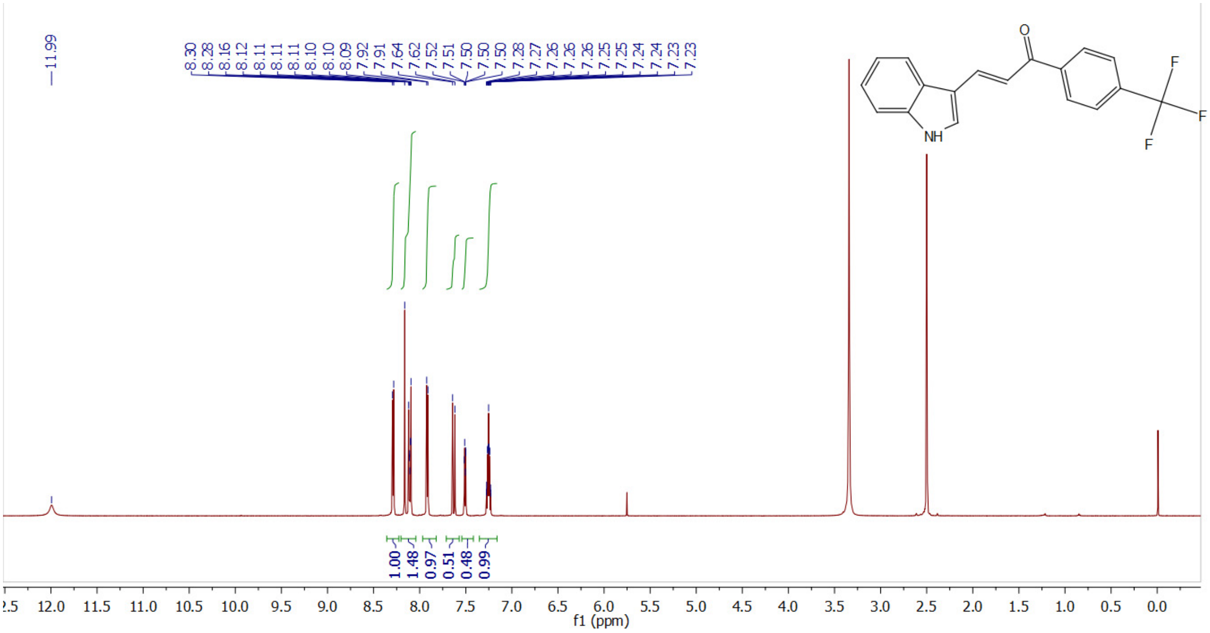

Figure S19: ^{1}H NMR spectra of compound **12a**

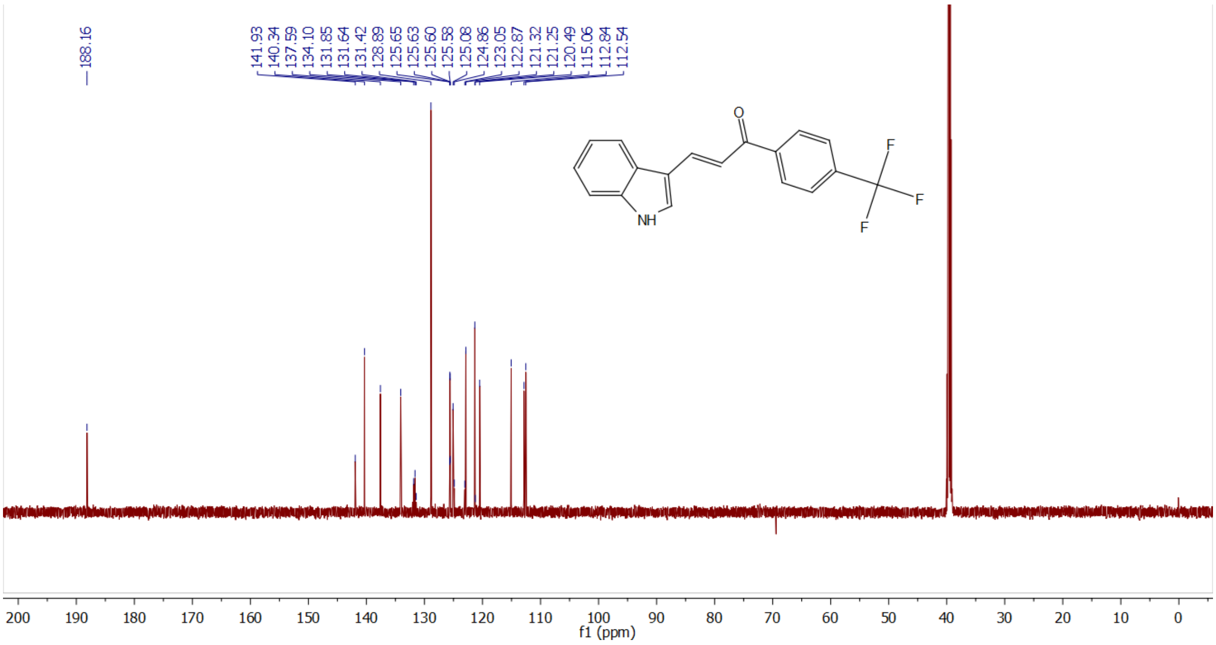

Figure S20: ^{13}C NMR spectra of compound **12a**

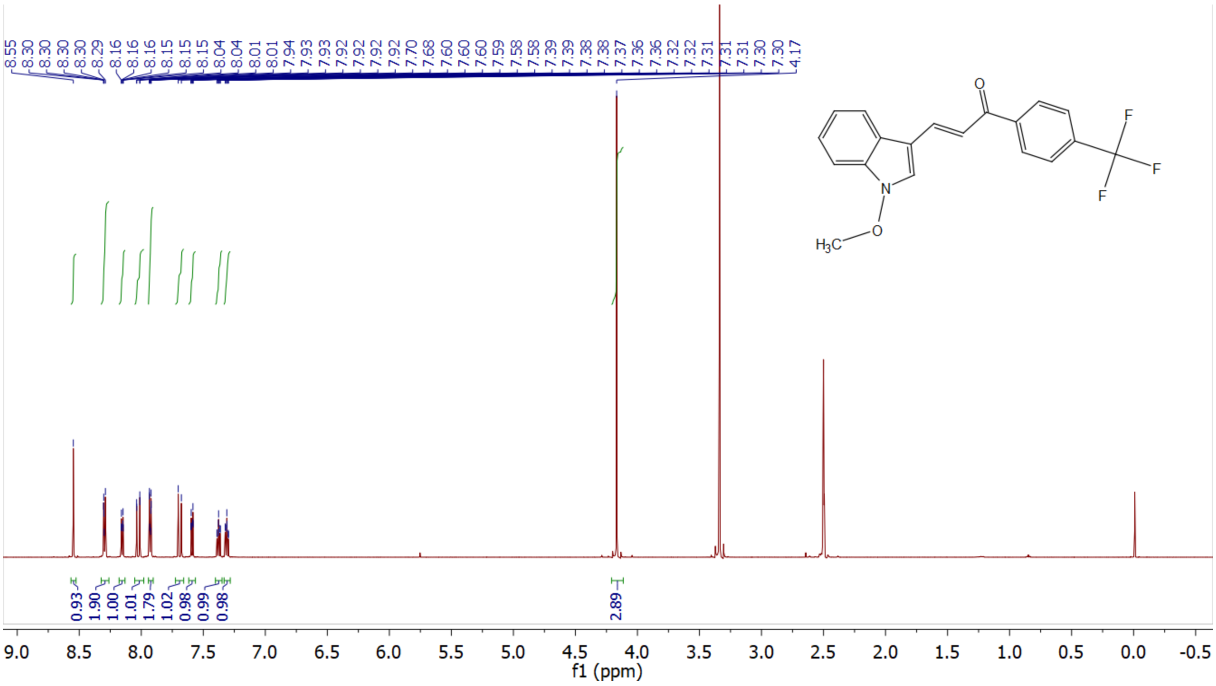

Figure S21: ^{1}H NMR spectra of compound **12c**

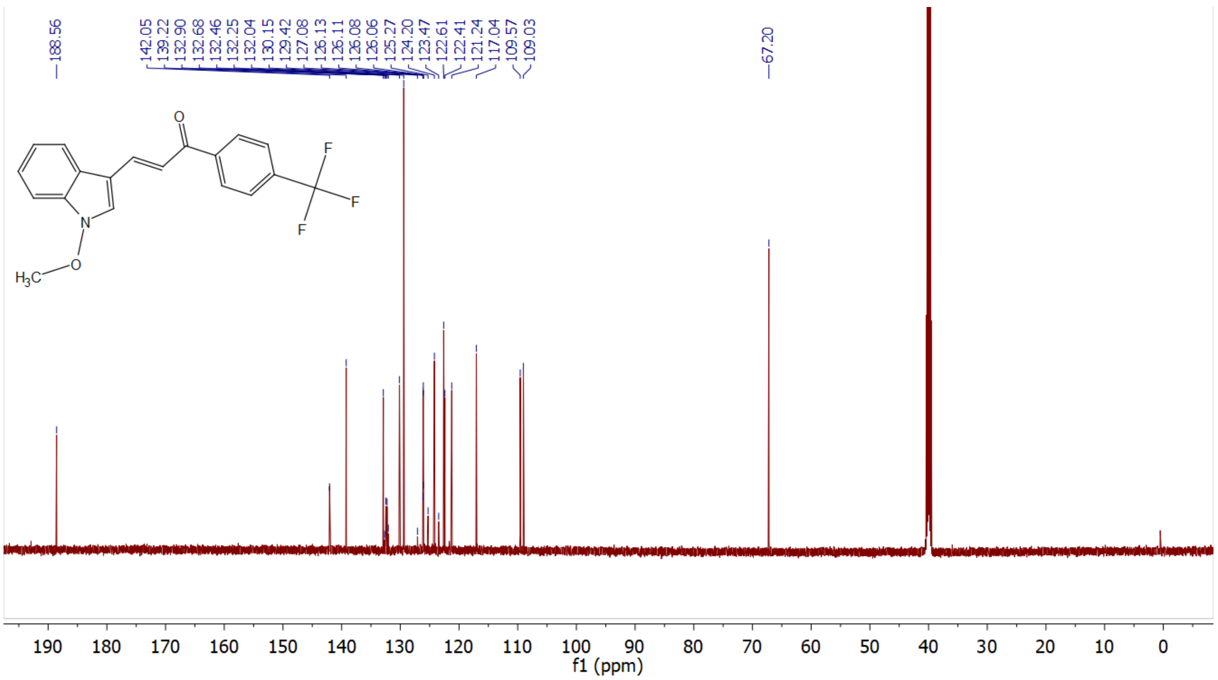

Figure S22: ^{13}C NMR spectra of compound **12c**

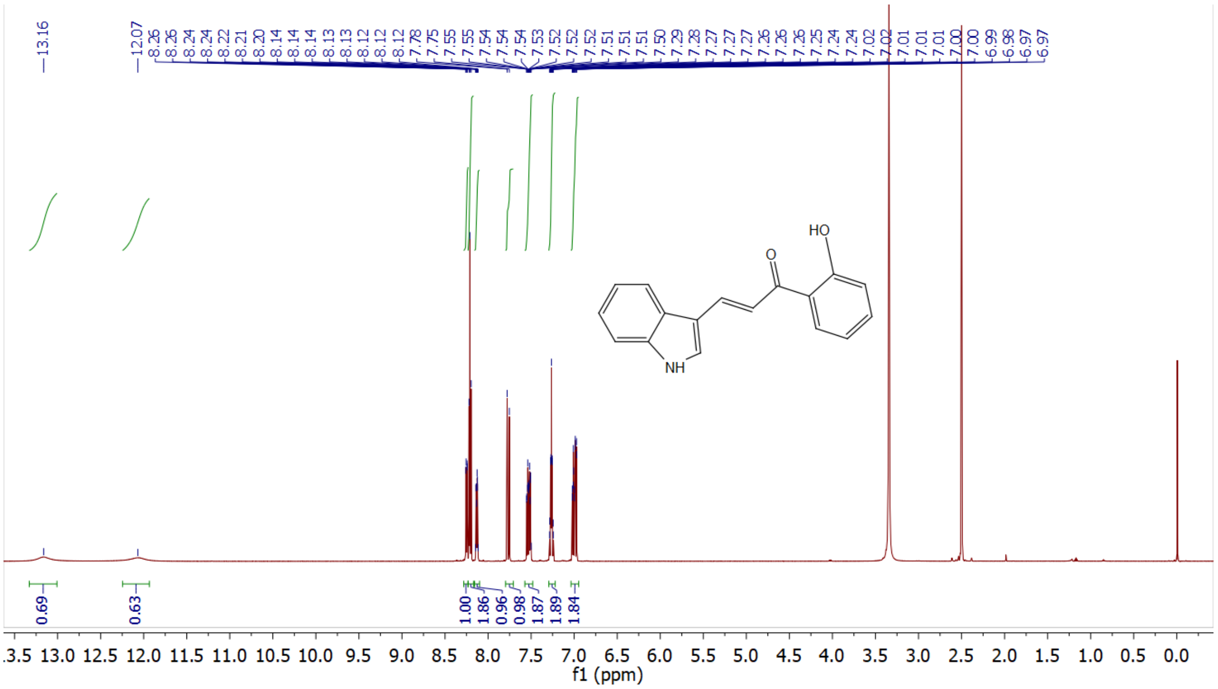

Figure S23: ^{1}H NMR spectra of compound **13a**

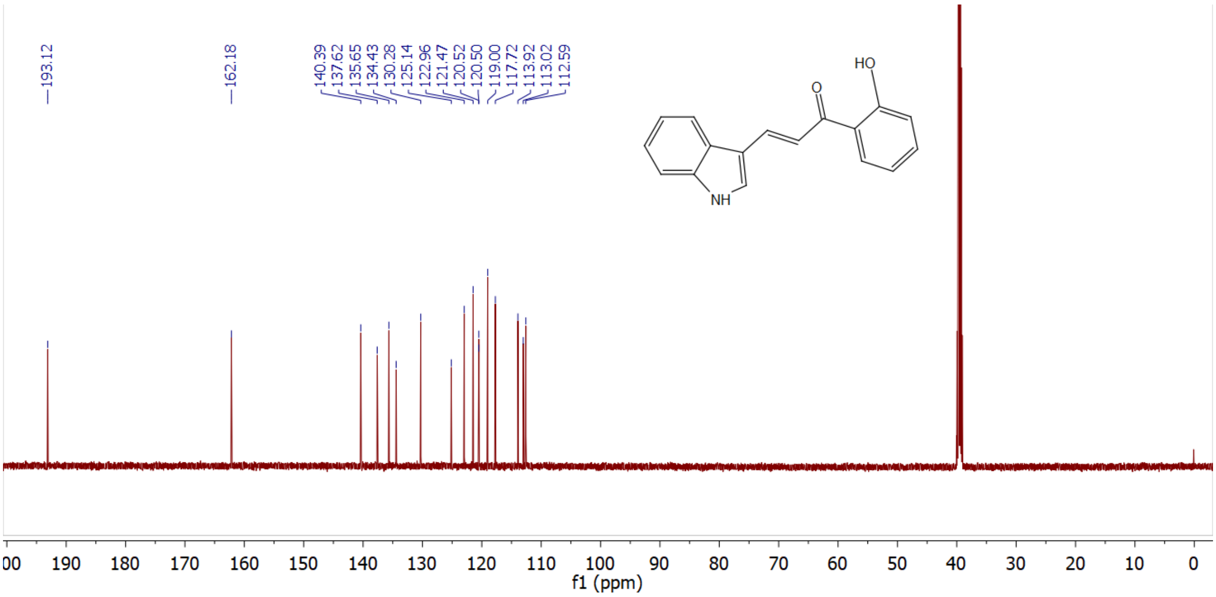

Figure S24: ^{13}C NMR spectra of compound **13a**

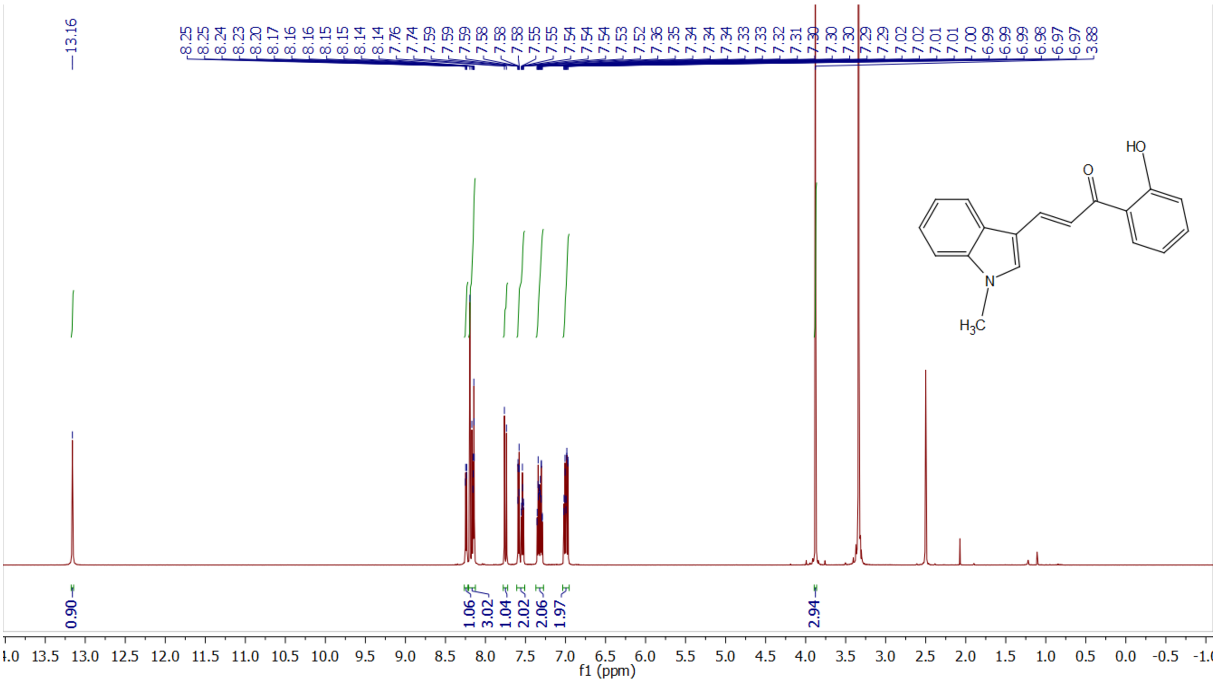

Figure S25: ^{1}H NMR spectra of compound **13b**

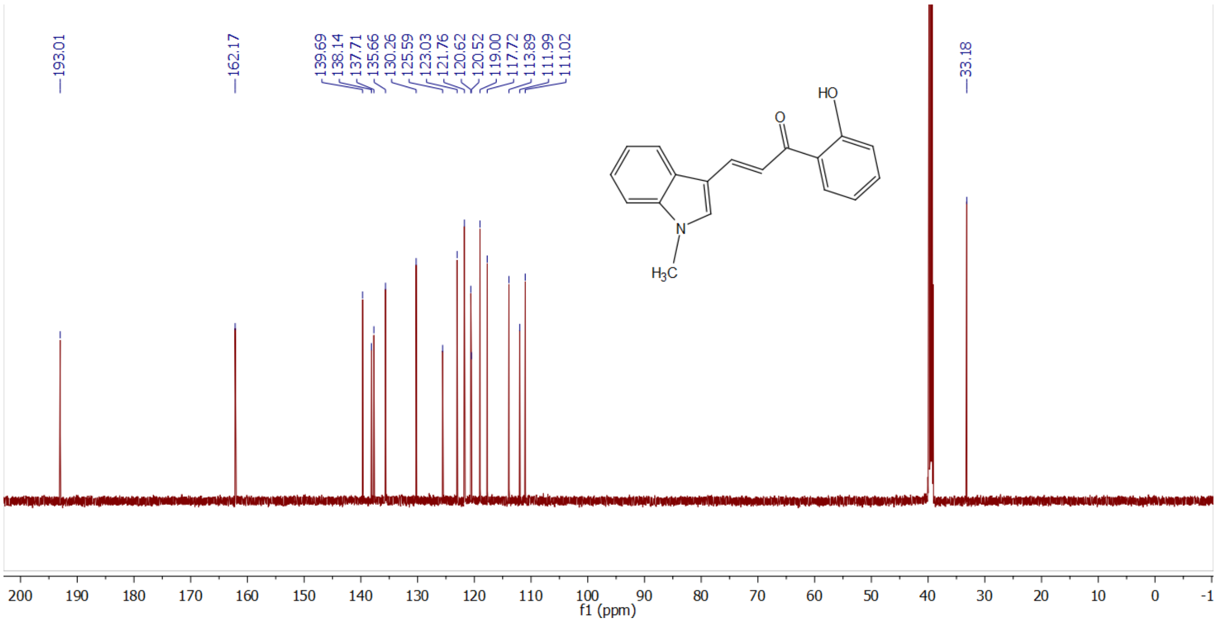

Figure S26: ^{13}C NMR spectra of compound **13b**

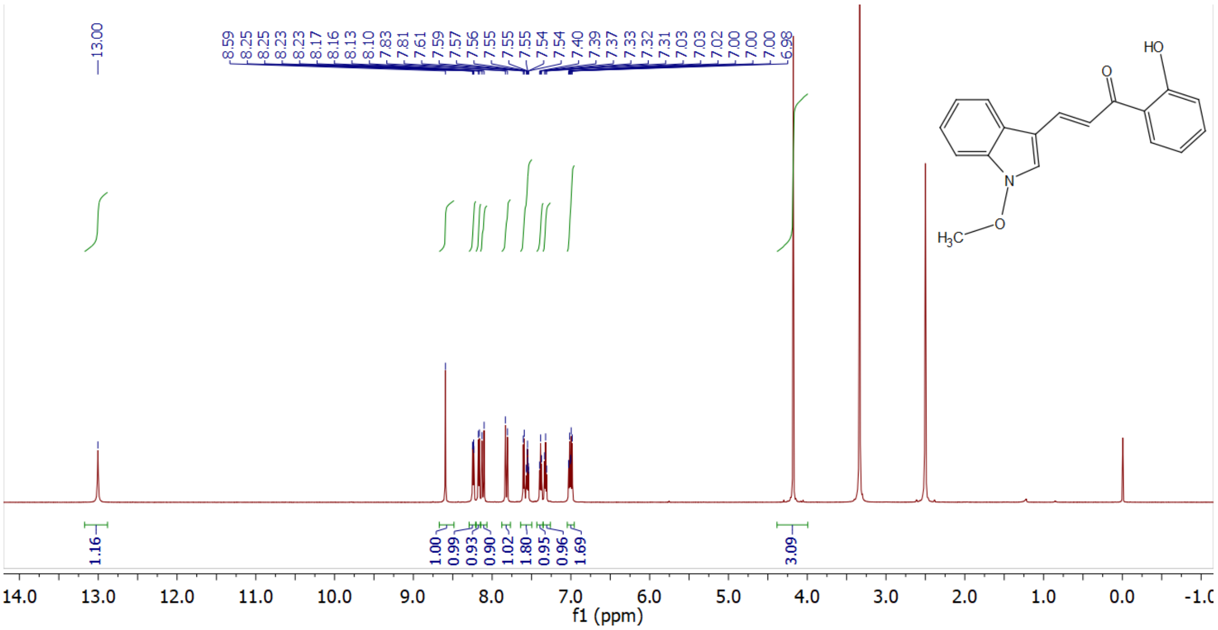

Figure S27: ^{1}H NMR spectra of compound **13c**

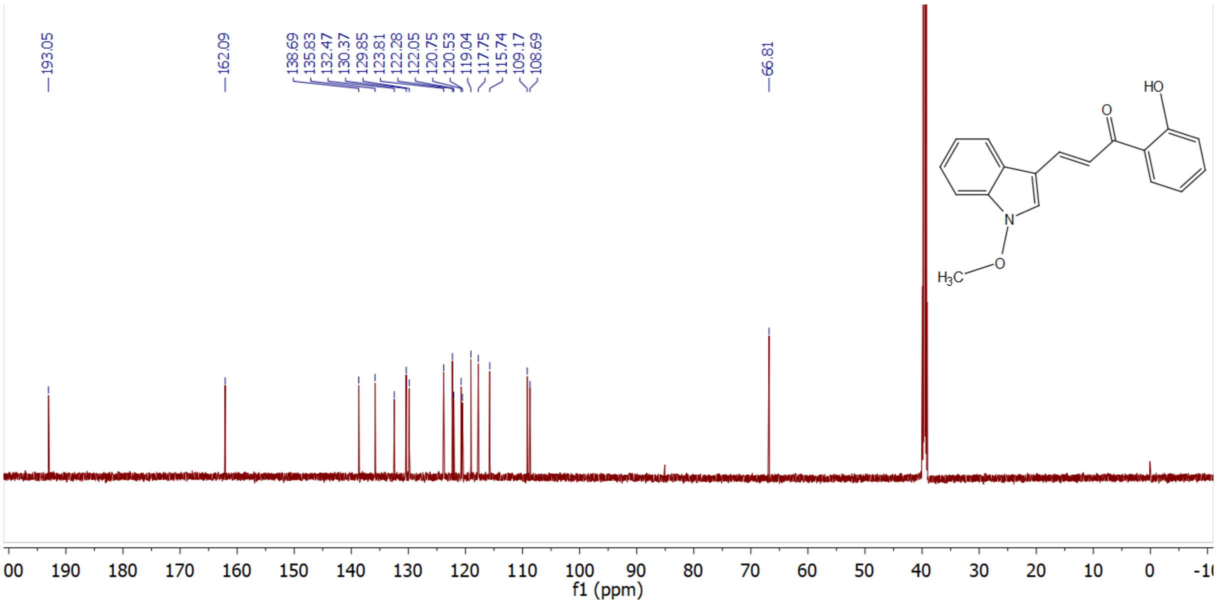

Figure S28: ^{13}C NMR spectra of compound **13c**

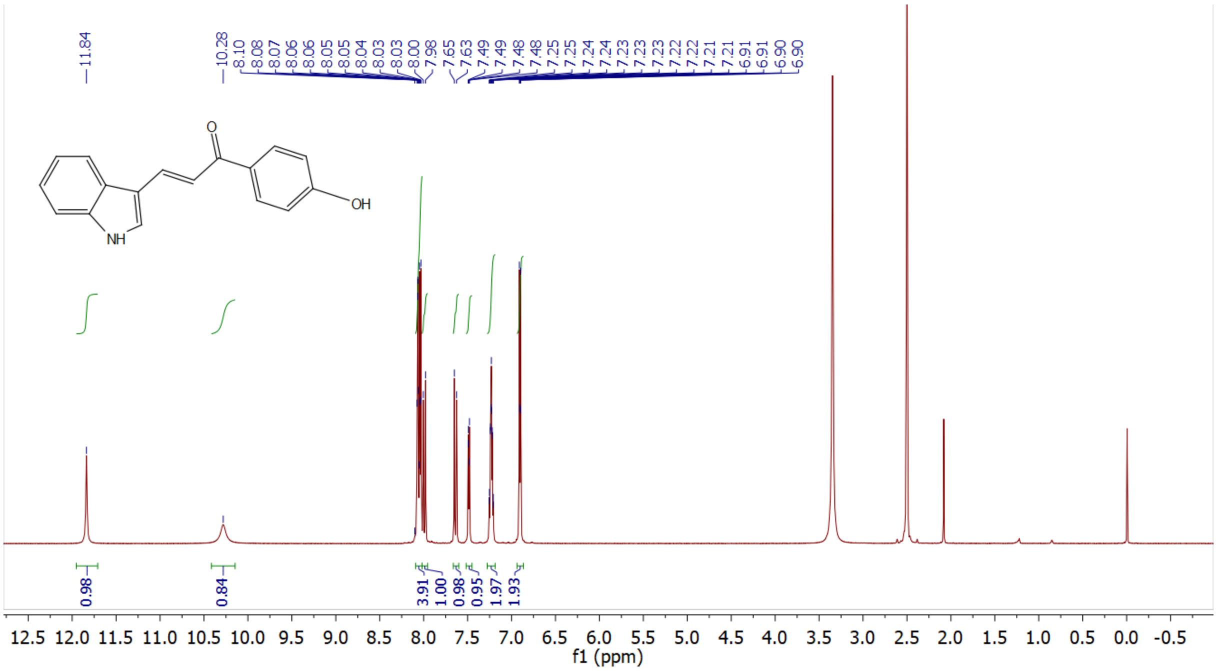

Figure S29: ^{1}H NMR spectra of compound **14a**

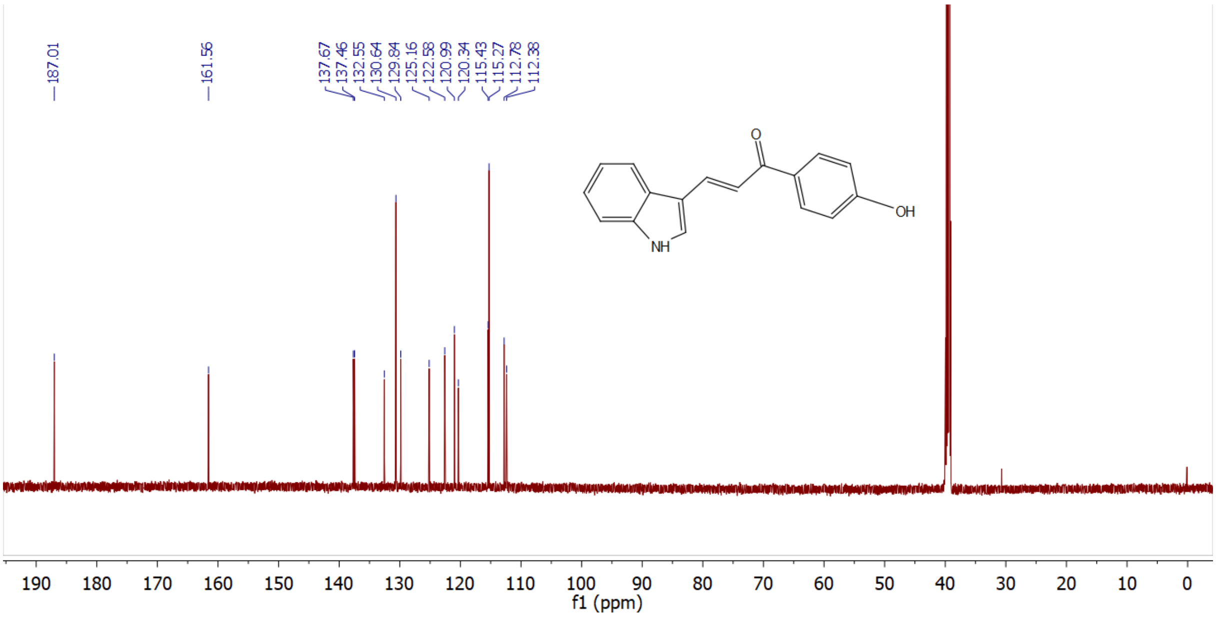

Figure S30: ^{13}C NMR spectra of compound **14a**

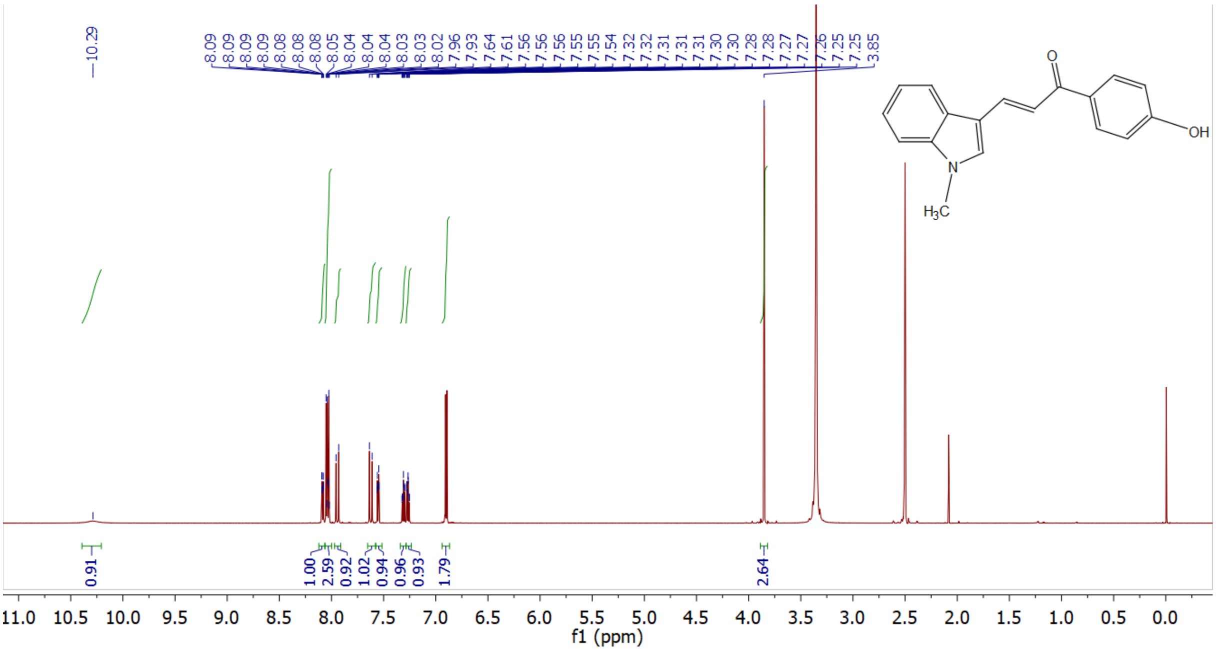

Figure S31: ^{1}H NMR spectra of compound **14b**

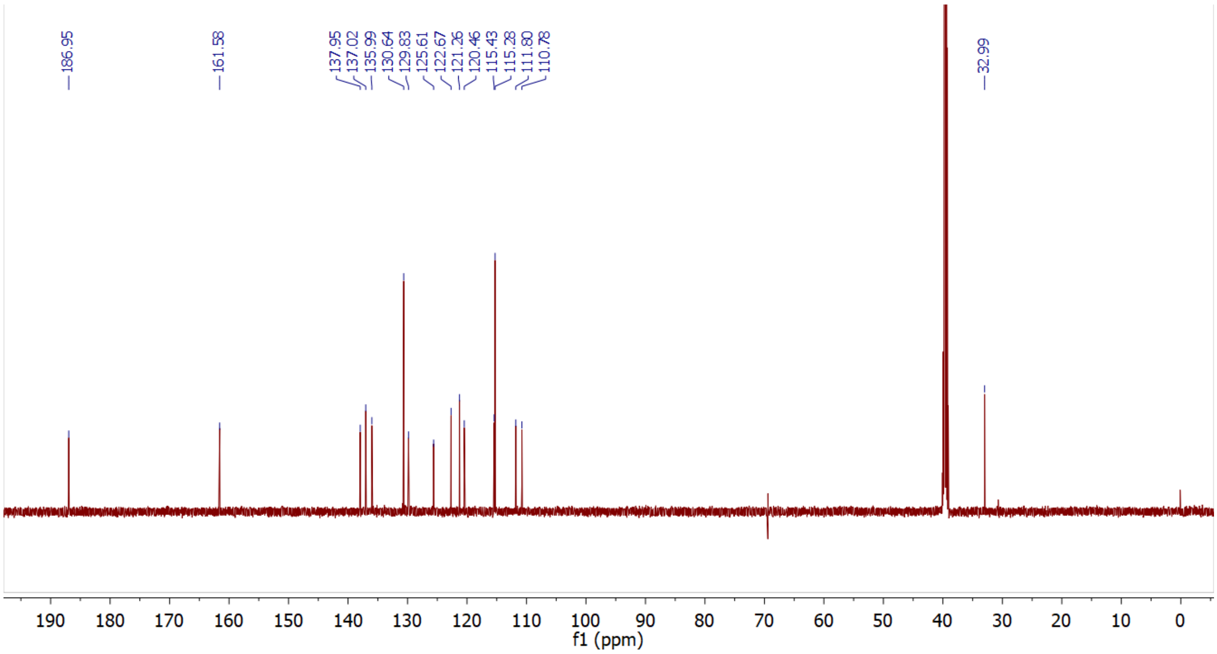

Figure S32: ^{13}C NMR spectra of compound **14b**

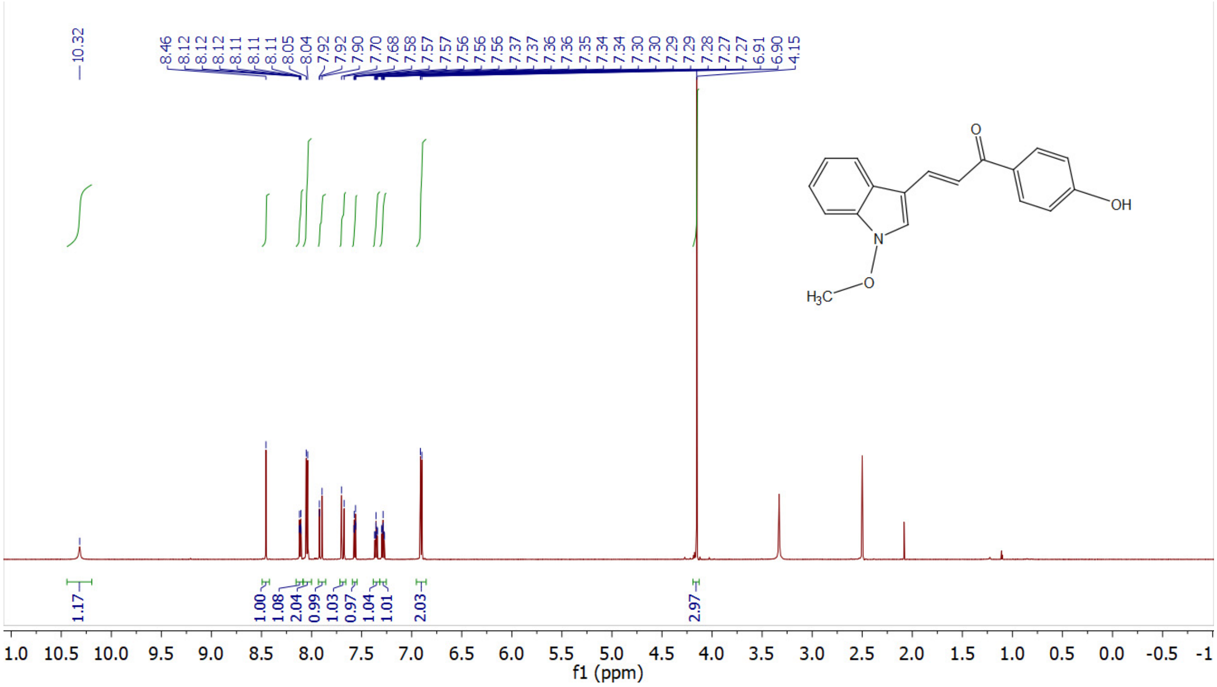

Figure S33: ^{1}H NMR spectra of compound **14c**

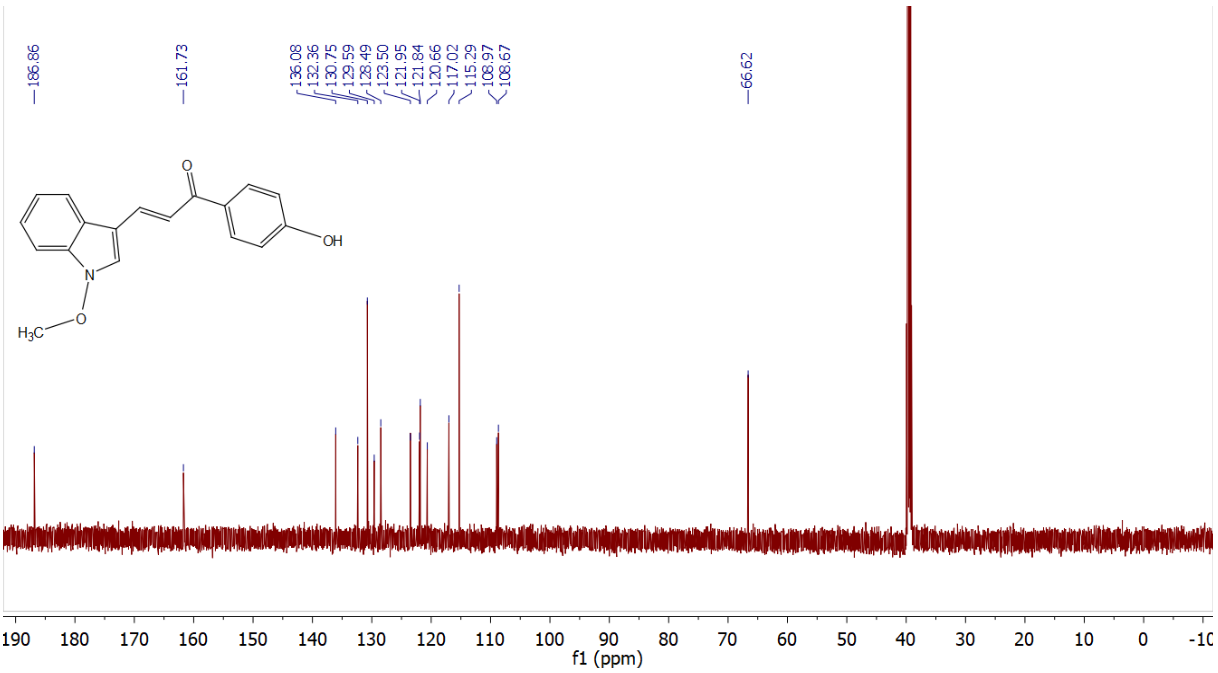

Figure S34: ^{13}C NMR spectra of compound **14c**

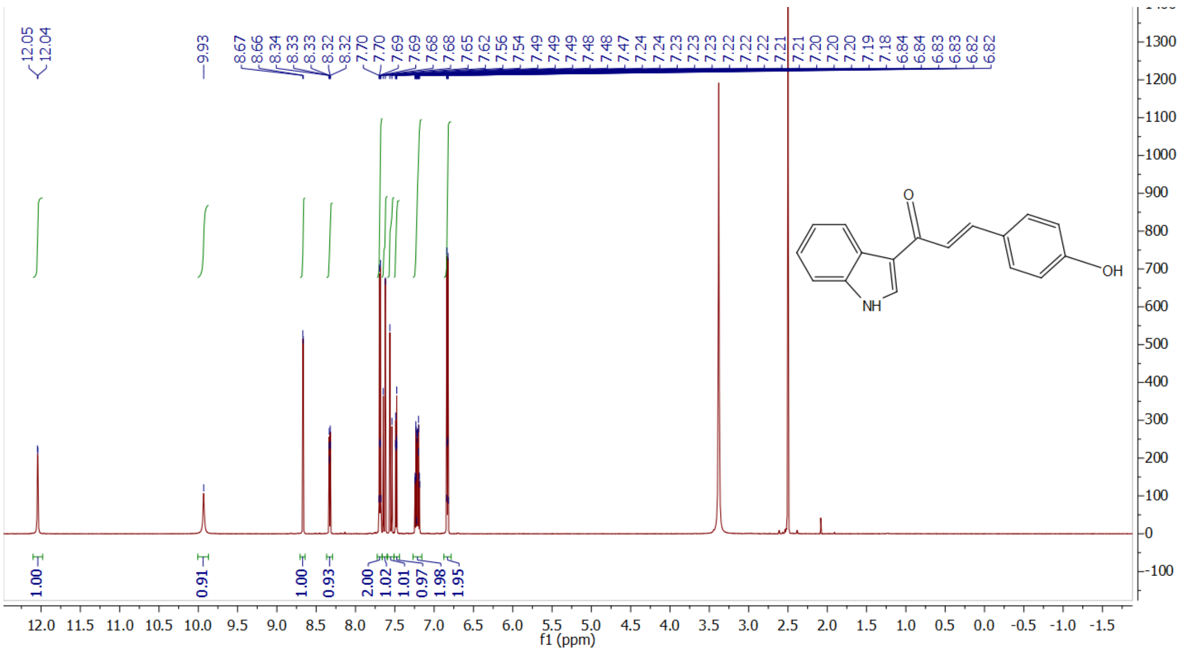

Figure S35: ^{1}H NMR spectra of compound **17a**

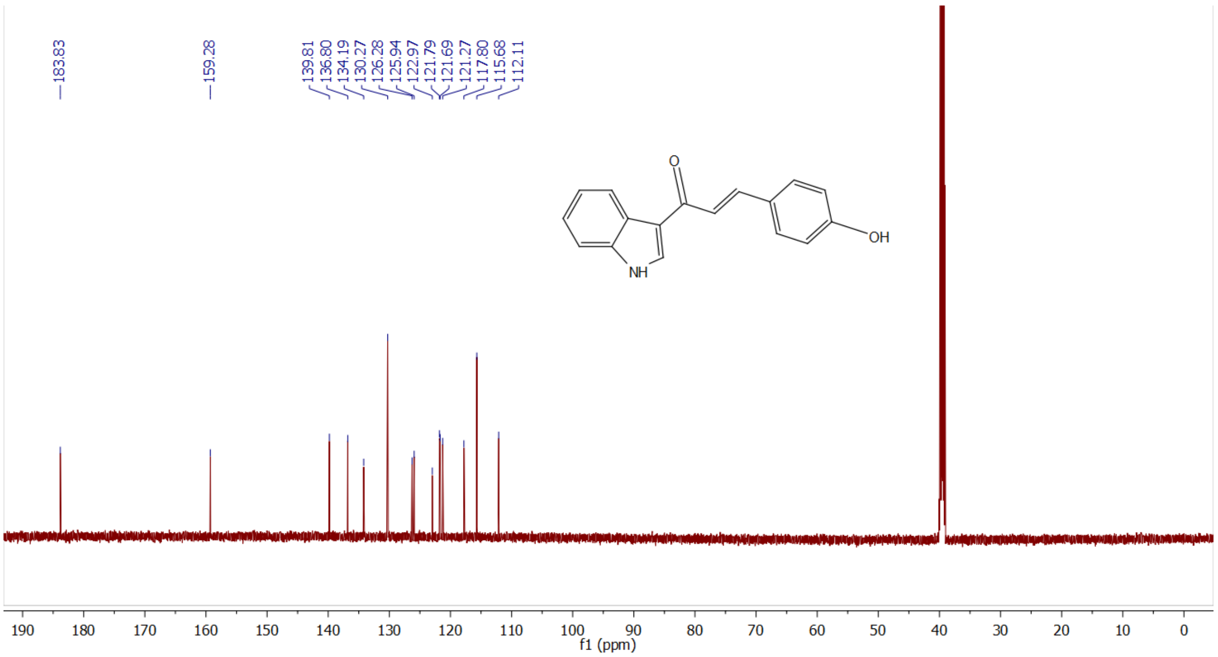

Figure S36: ^{13}C NMR spectra of compound **17a**

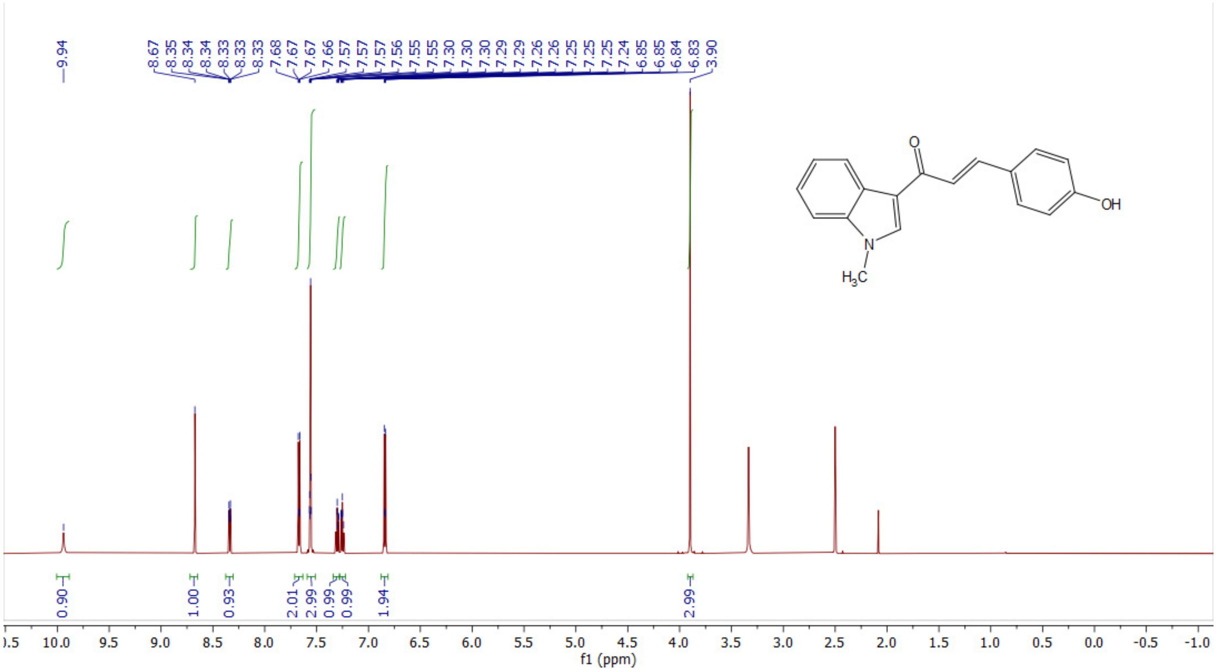

Figure S37: ^{1}H NMR spectra of compound **17b**

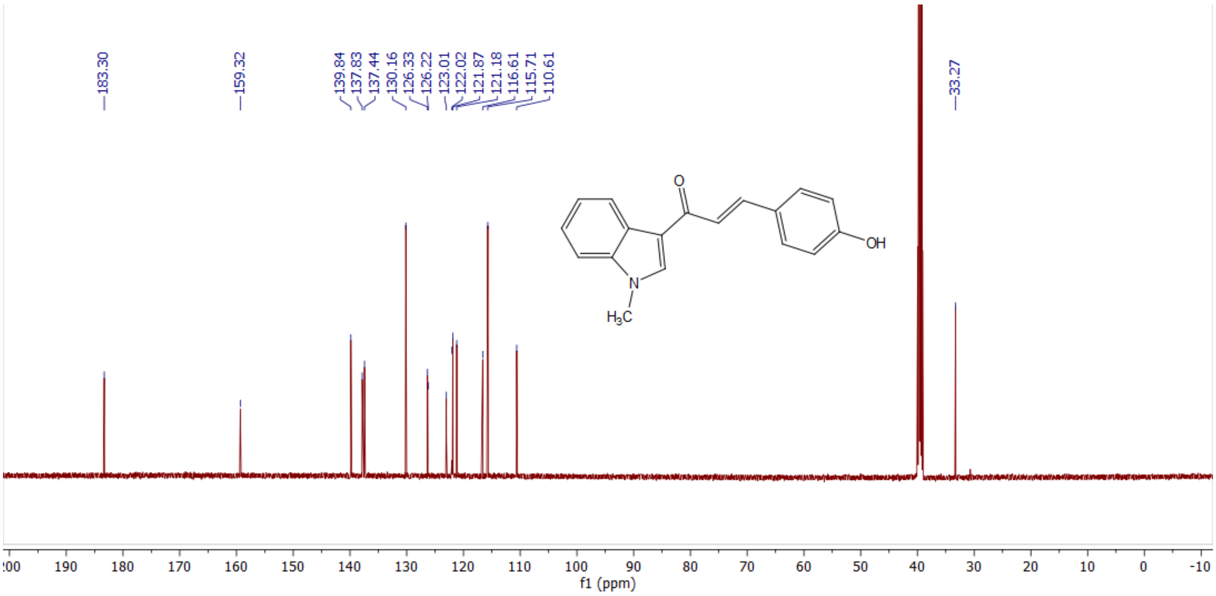

Figure S38: ^{13}C NMR spectra of compound **17b**

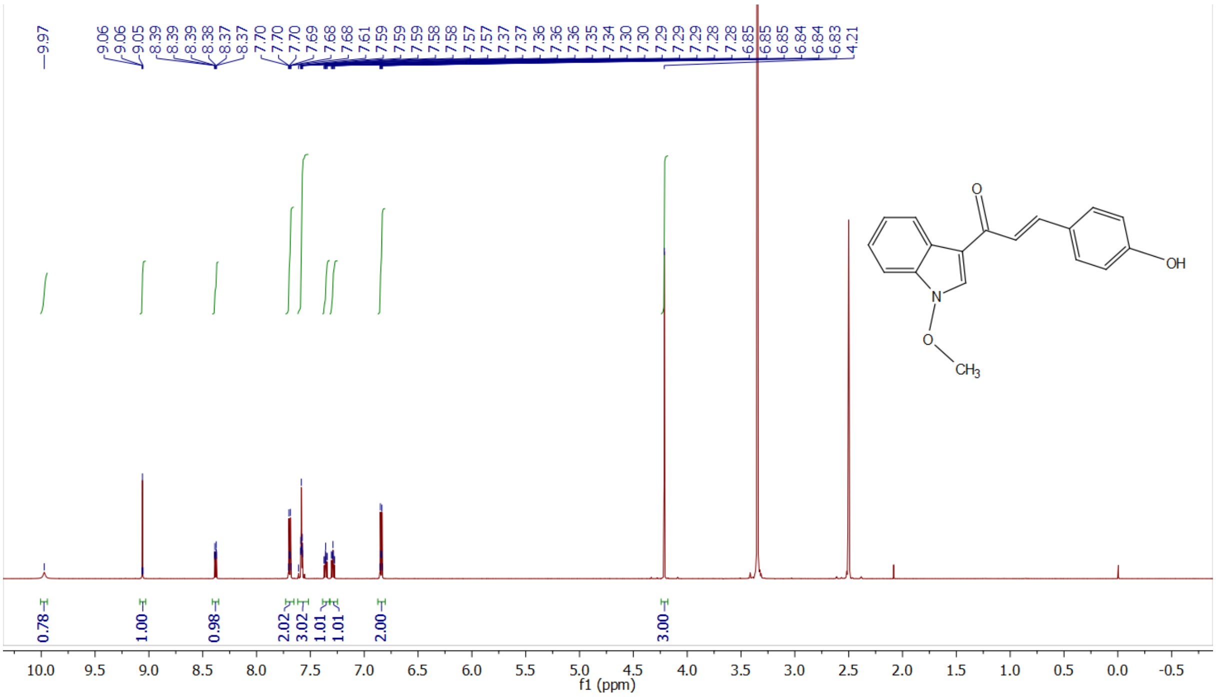

Figure S39: ^{1}H NMR spectra of compound **17c**

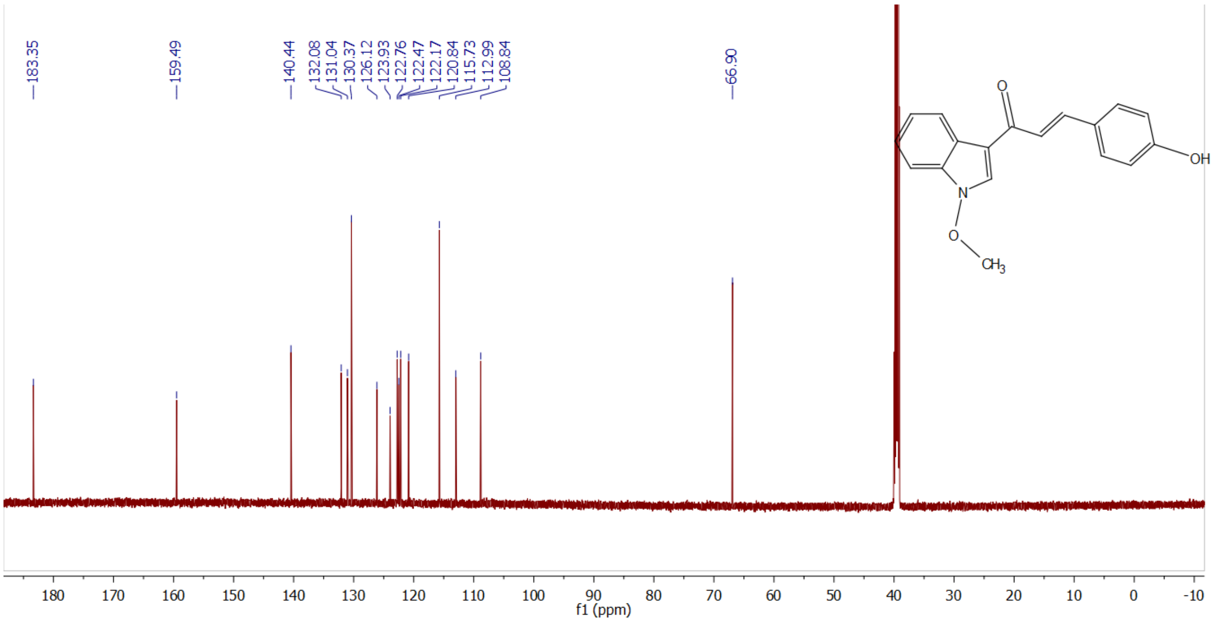

Figure S40: ^{13}C NMR spectra of compound **17c**

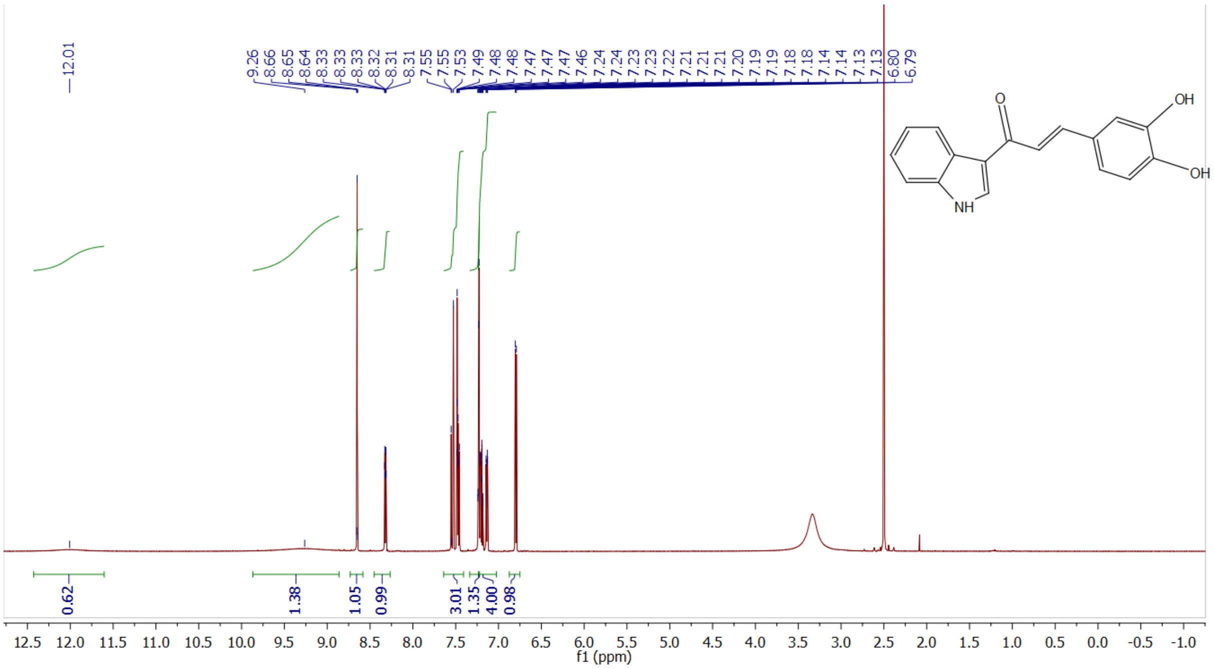

Figure S41: ^{1}H NMR spectra of compound **18a**

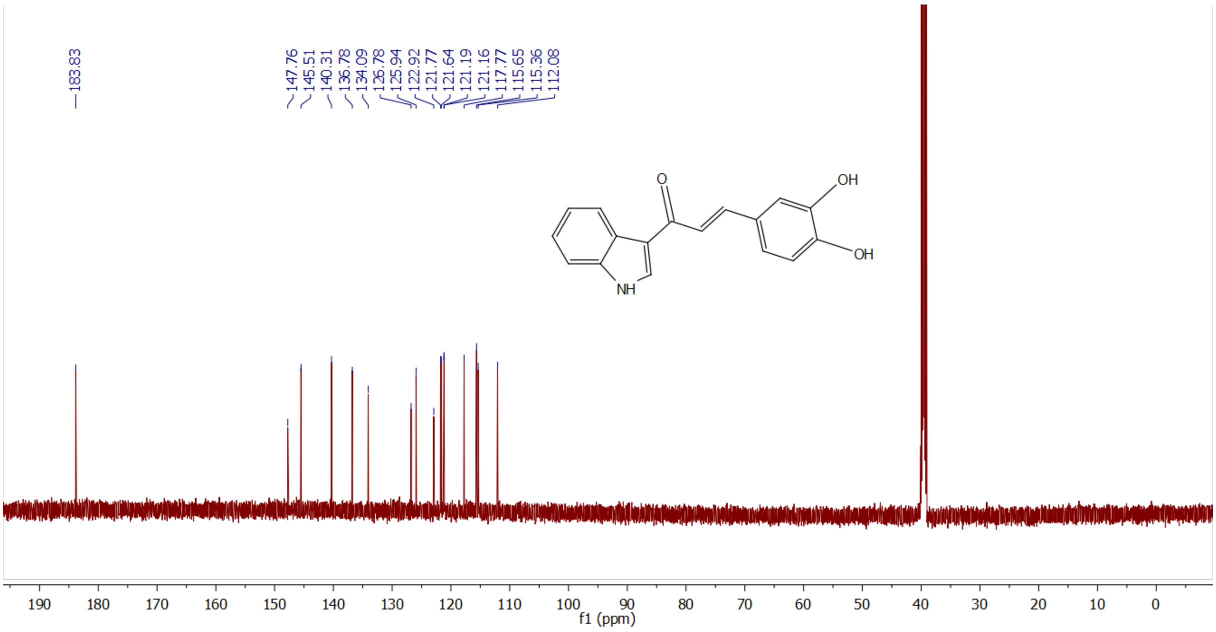

Figure S42: ^{13}C NMR spectra of compound **18a**

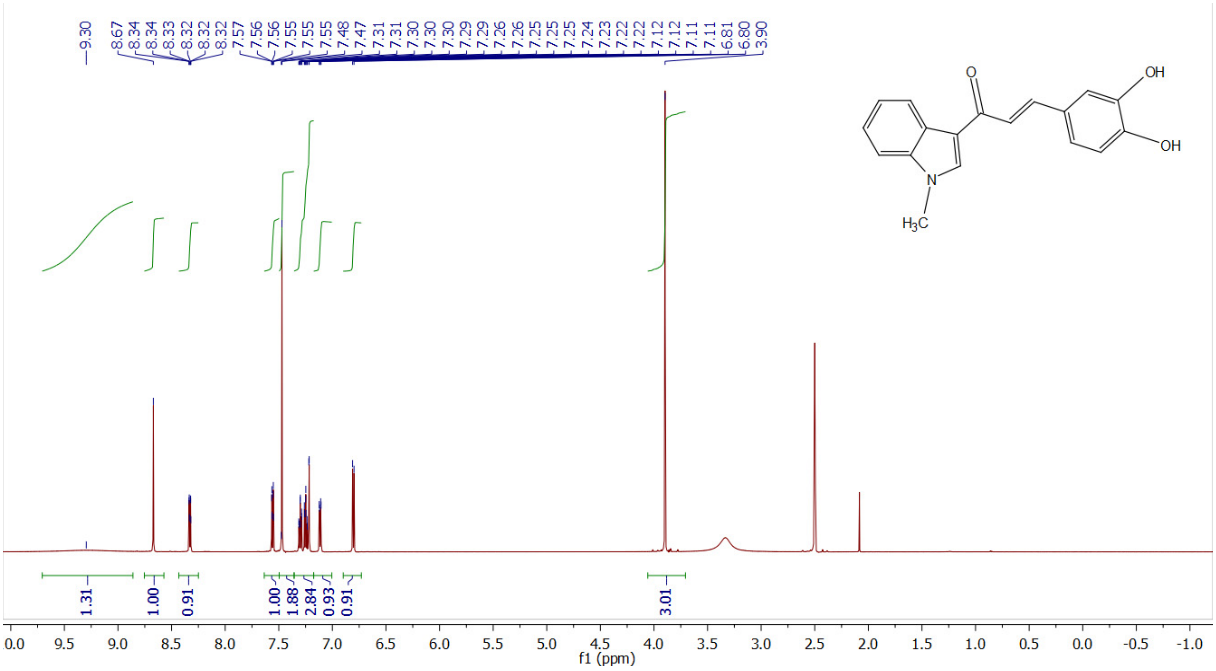

Figure S43: ^{1}H NMR spectra of compound **18b**

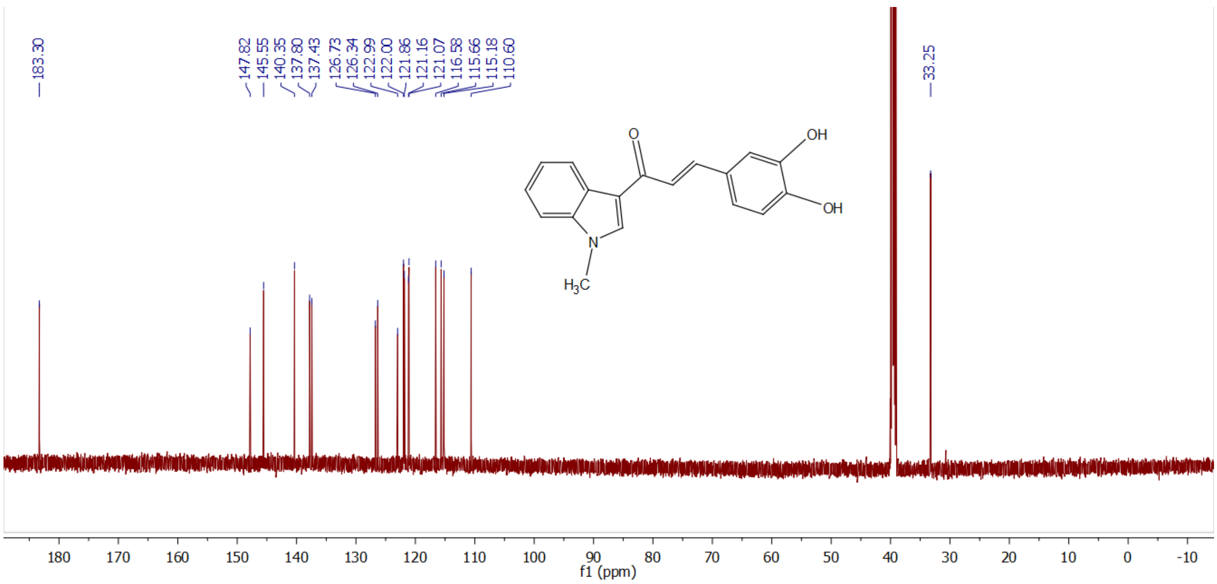

Figure S44: ^{13}C NMR spectra of compound **18b**

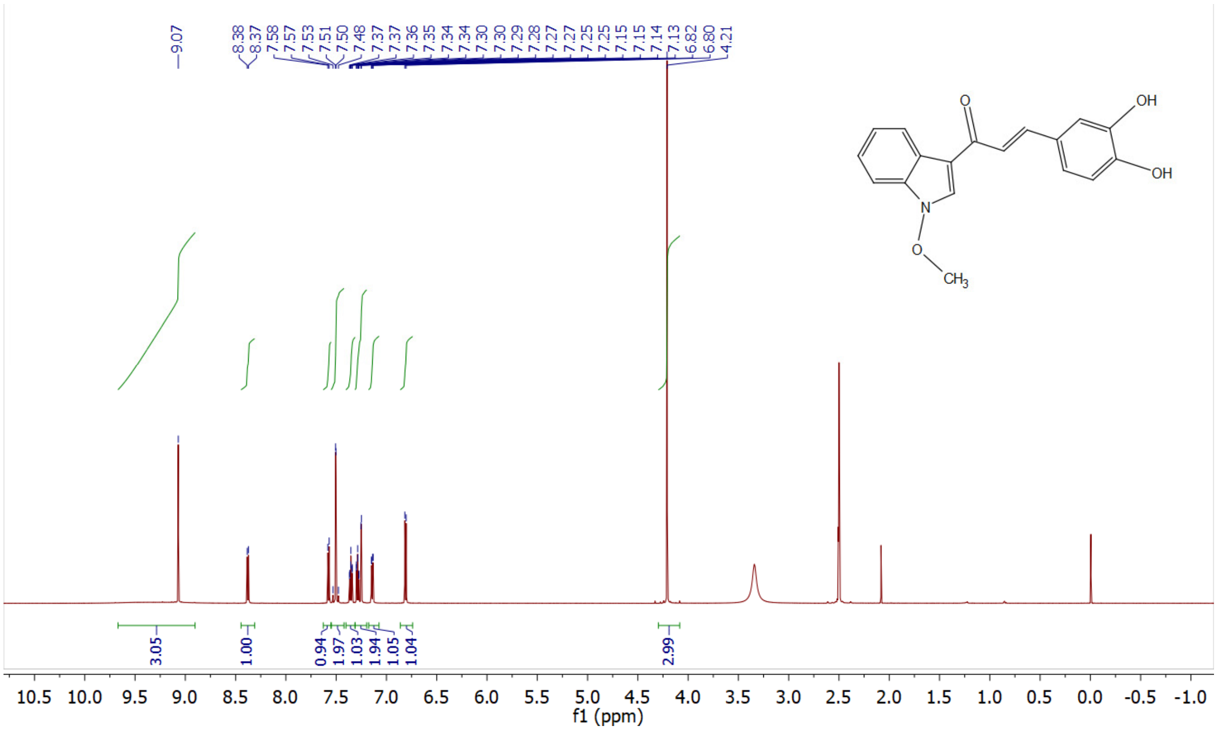

Figure S45: ^{1}H NMR spectra of compound **18c**

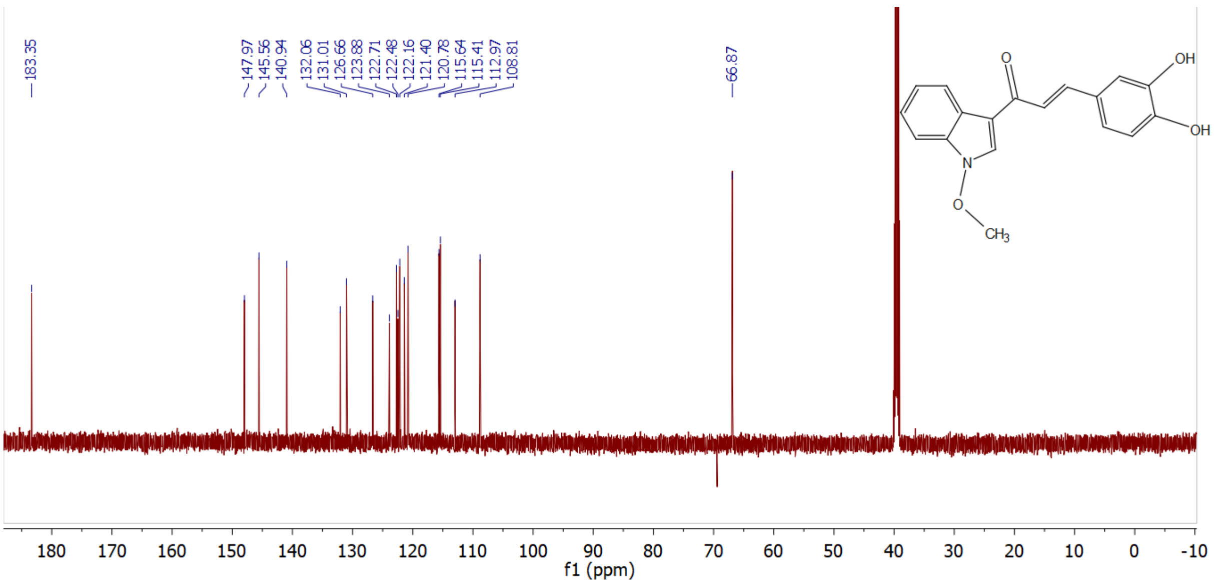

Figure S46: ^{13}C NMR spectra of compound **18c**

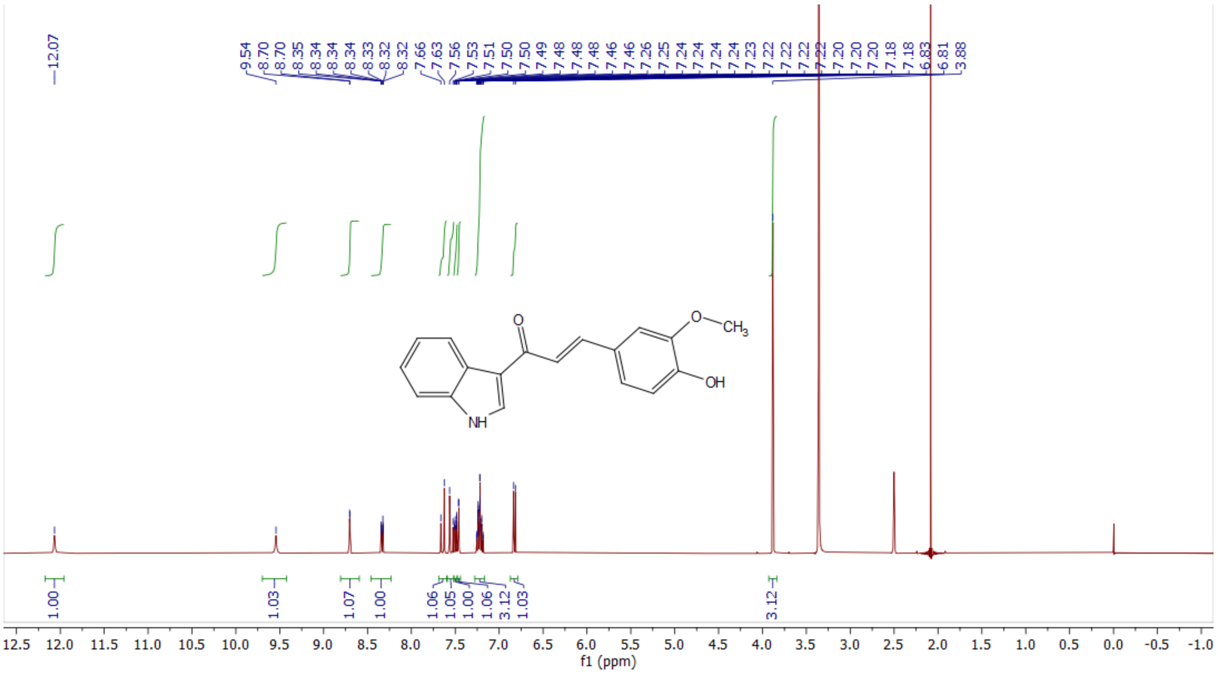

Figure S47: ^{1}H NMR spectra of compound **19a**

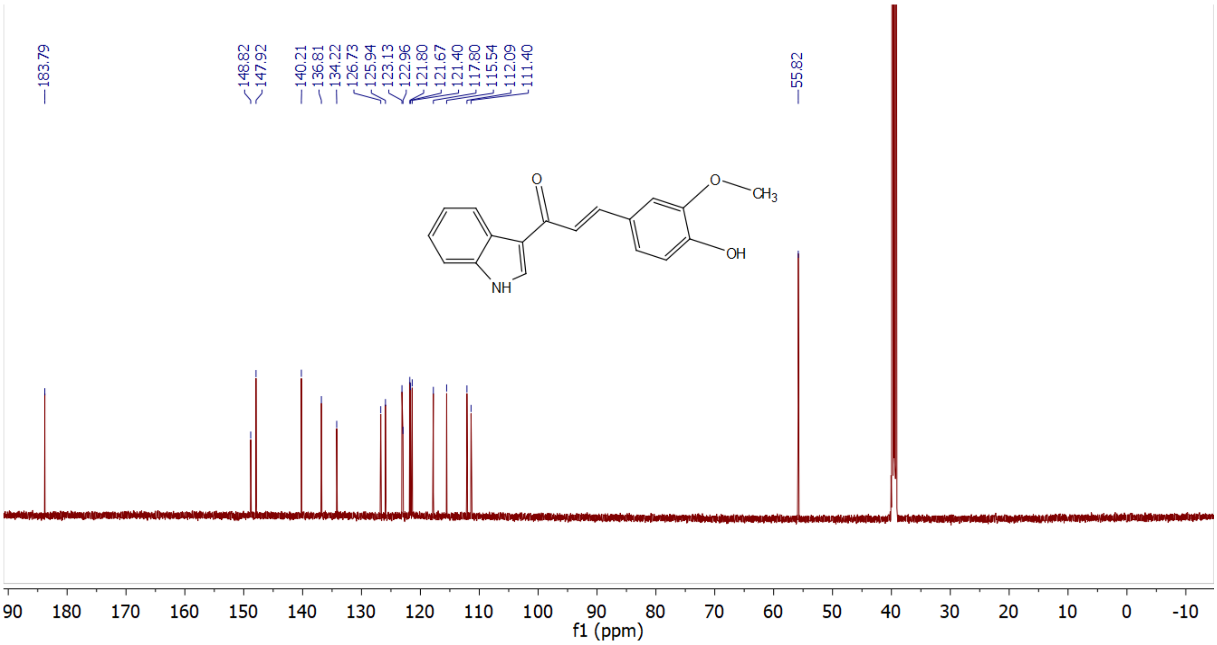

Figure S48: ^{13}C NMR spectra of compound **19a**

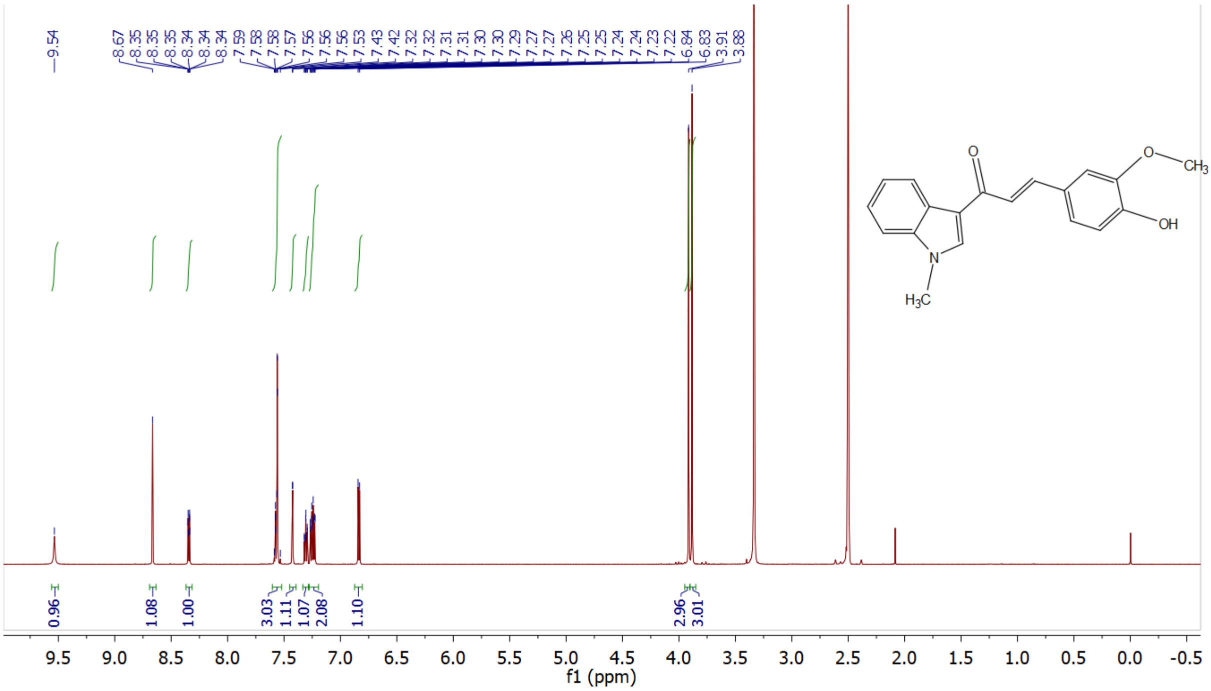

Figure S49: ^{1}H NMR spectra of compound **19b**

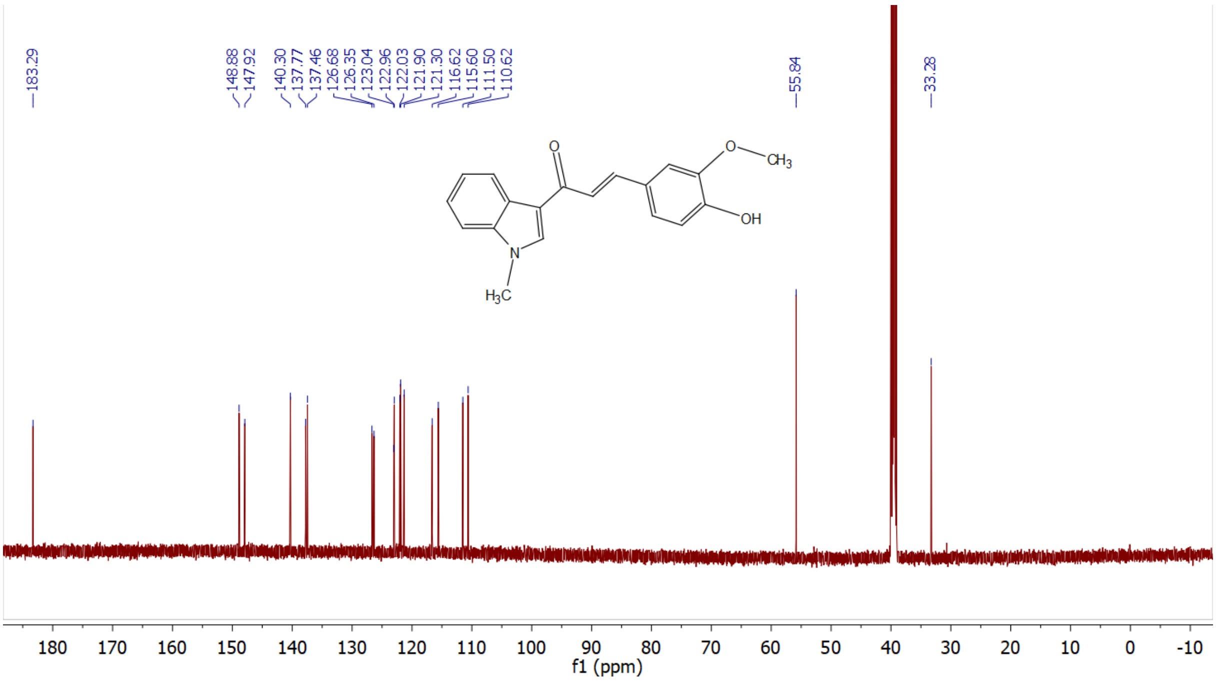

Figure S50: ^{13}C NMR spectra of compound **19b**

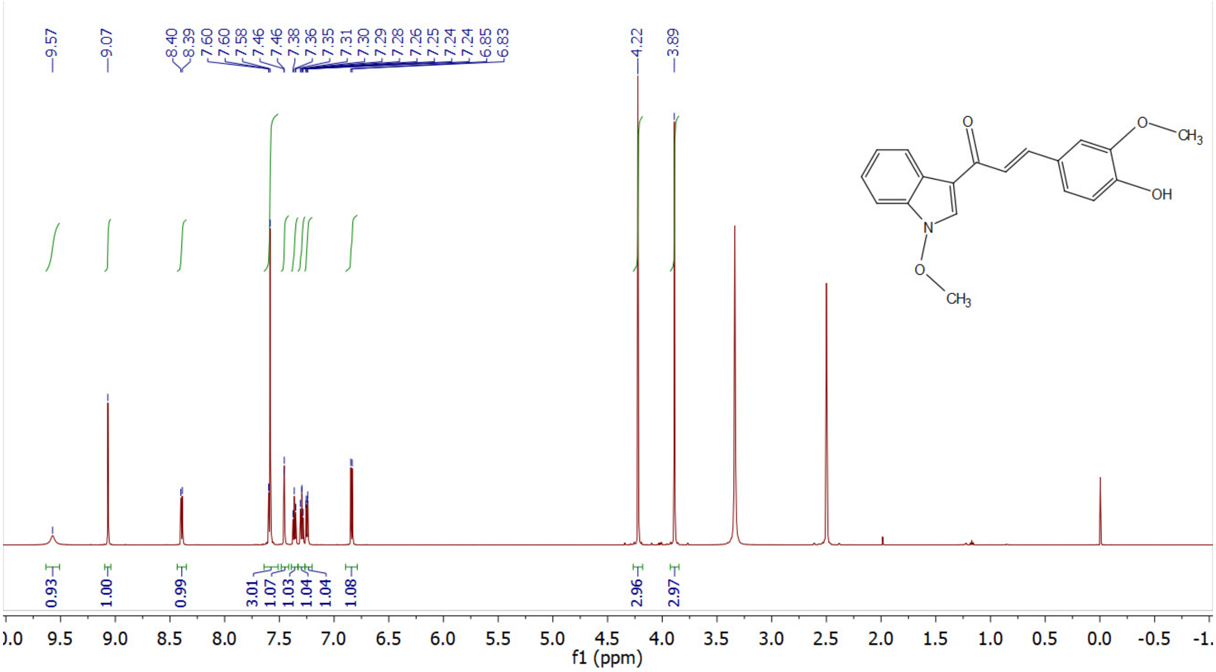

Figure S51: ^{1}H NMR spectra of compound **19c**

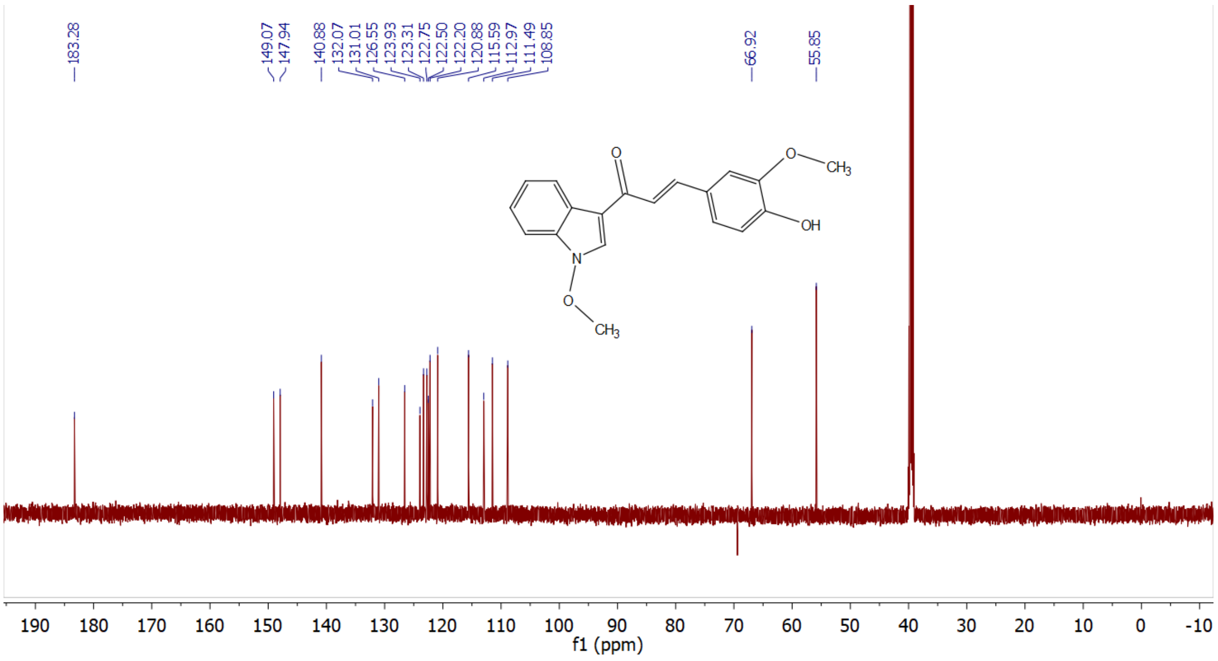

Figure S52: ^{13}C NMR spectra of compound **19c**

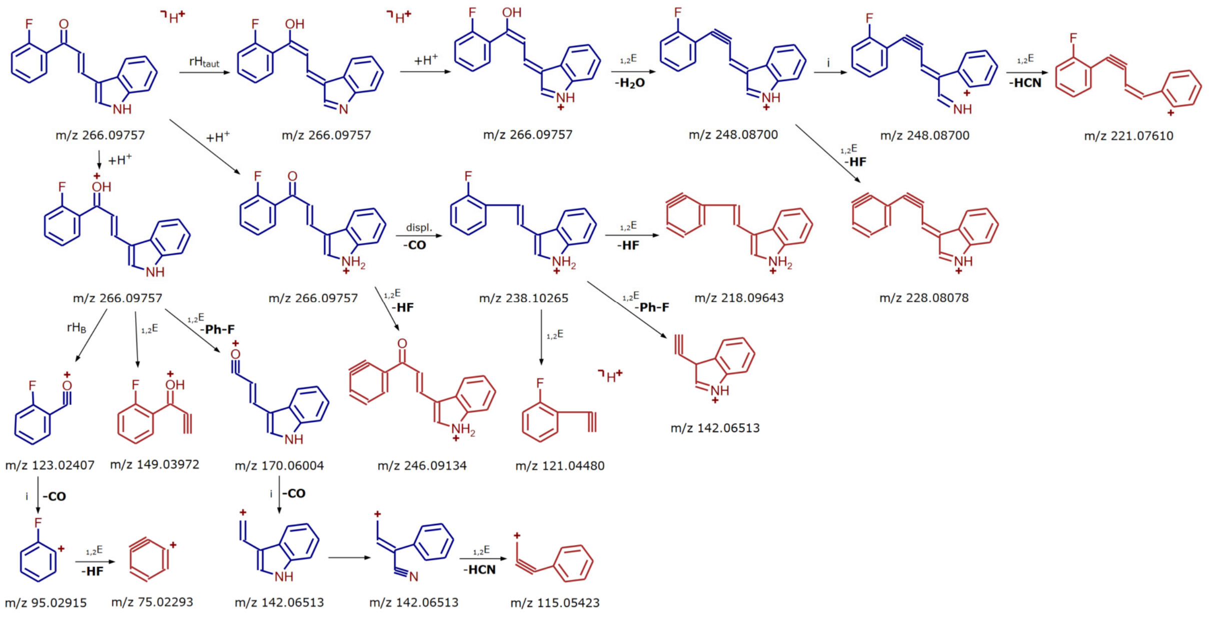

Figure S53: HR-MS fragmentation spectra for compound **11a**
